# Intestinal microbiota by angiotensin receptor blocker therapy exerts protective effects against hypertensive damages

**DOI:** 10.1002/imt2.222

**Published:** 2024-07-18

**Authors:** Jing Li, Si‐Yuan Wang, Kai‐Xin Yan, Pan Wang, Jie Jiao, Yi‐Dan Wang, Mu‐Lei Chen, Ying Dong, Jiu‐Chang Zhong

**Affiliations:** ^1^ Heart Center and Beijing Key Laboratory of Hypertension, Beijing Chaoyang Hospital Capital Medical University Beijing China; ^2^ Department of Cardiology, Beijing Chaoyang Hospital Capital Medical University Beijing China

**Keywords:** angiotensin receptor blockers, antihypertensive, gut microbiota, hypertension, vascular injury

## Abstract

Dysbiosis of the gut microbiota has been implicated in hypertension, and drug–host–microbiome interactions have drawn considerable attention. However, the influence of angiotensin receptor blocker (ARB)‐shaped gut microbiota on the host is not fully understood. In this work, we assessed the alterations of blood pressure (BP), vasculatures, and intestines following ARB‐modified gut microbiome treatment and evaluated the changes in the intestinal transcriptome and serum metabolome in hypertensive rats. Hypertensive patients with well‐controlled BP under ARB therapy were recruited as human donors, spontaneously hypertensive rats (SHRs) receiving normal saline or valsartan were considered animal donors, and SHRs were regarded as recipients. Histological and immunofluorescence staining was used to assess the aorta and small intestine, and 16S rRNA amplicon sequencing was performed to examine gut bacteria. Transcriptome and metabonomic analyses were conducted to determine the intestinal transcriptome and serum metabolome, respectively. Notably, ARB‐modified fecal microbiota transplantation (FMT), results in marked decreases in systolic BP levels, collagen deposition and reactive oxygen species accumulation in the vasculature, and alleviated intestinal structure impairments in SHRs. These changes were linked with the reconstruction of the gut microbiota in SHR recipients post‐FMT, especially with a decreased abundance of *Lactobacillus*, *Aggregatibacter*, and *Desulfovibrio*. Moreover, ARB‐treated microbes contributed to increased intestinal *Ciart*, *Per1*, *Per2*, *Per3*, and *Cipc* gene levels and decreased *Nfil3* and *Arntl* expression were detected in response to ARB‐treated microbes. More importantly, circulating metabolites were dramatically reduced in ARB‐FMT rats, including 6beta‐Hydroxytestosterone and Thromboxane B2. In conclusion, ARB‐modified gut microbiota exerts protective roles in vascular remodeling and injury, metabolic abnormality and intestinal dysfunctions, suggesting a pivotal role in mitigating hypertension and providing insights into the cross‐talk between antihypertensive medicines and the gut microbiome.

## Highlights

- Angiotensin receptor blocker (ARB)‐modified gut microbiota leads to reduced systolic blood pressure levels and exerts protective roles in spontaneously hypertensive rats (SHRs).
- ARB‐modified fecal microbiota transplantation (FMT) contributes to the reconstruction of the gut microbiota, serum metabolome, and intestinal transcriptome SHRs.
- Treatment with ARB‐modified FMT results in marked decreases in collagen deposition and reactive oxygen species accumulation in the vasculature as well as alleviated vascular and intestinal structural impairments in hypertensive rats.

## Graphical abstract

16S rRNA sequencing, liquid chromatography‐tandem mass spectrometry, RNA sequencing and immunofluorescence staining were combined to assess the role of ARB‐modified gut microbes. ARB‐modified gut microbiota exert protective roles in hypertension, vascular remodeling and injury, metabolic abnormality and intestinal dysfunctions, suggesting a pivotal role in mitigating hypertension and providing insights into the cross‐talk between antihypertensive medicines and the gut microbiome.

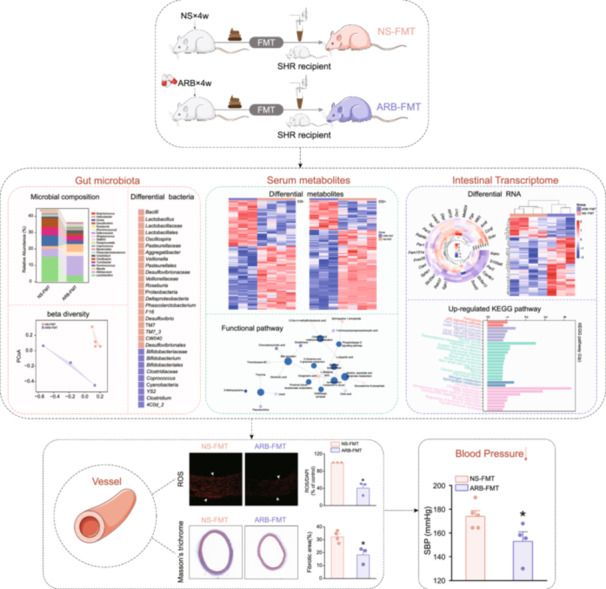

## INTRODUCTION

Hypertension is one of the most prevalent chronic diseases with more than 1.28 billion people worldwide and has been directly associated with up to 8.5 million deaths per year. It is also the most important risk factor for many malignant conditions, including stroke, ischemic heart disease, and renal dysfunction [1]. Globally, hypertension is responsible for approximately 70% of strokes, 50% of acute myocardial infarction, 50% of atrial fibrillation, and 20%–30% of renal insufficiency [2]. Currently, the prevalence of hypertension in Chinese residents aged ≥18 was 25.2%, but only 15.3% showed well‐controlled (WC) hypertension [3]. The pathogenesis of hypertension is complex. Accumulating evidence shows that the intestinal flora has an essential role in development of hypertension [4, 5]. Disordered intestinal bacteria may lead to elevated blood pressure (BP) and damage the heart, vessels, and kidneys [6]. For instance, *Lactobacillus spp.* was observed in individuals with lower BP variability [7]. It has been demonstrated that a Western lifestyle characterized by high salt intake contributes to hypertension by depleting *Lactobacillus murinus* [8]. Notably, species from *Aggregatibacter* were found to be significantly elevated in preeclamptic women with hypertension plus proteinuria, and the enrichment of *Aggregatibacter* in spontaneously hypertensive animals could be depressed by exercise [9, 10]. Moreover, *Desulfovibrio*, an opportunistic pathogen, was observed to be positively correlated with systolic BP (SBP), and its association with a high risk of adverse cardiovascular effects has been previously explained [11, 12, 13]. Intriguingly, modulating the gut microbiome to maintain homeostasis or regulate its metabolites may facilitate the improvement of the host's BP. Recent studies have shown that exercise can reshape the intestinal flora, reduce BP, suppress neuroinflammation, and improve intestinal pathology and intestinal mucosal permeability in hypertensive rats [14].

Patients with hypertension frequently receive treatment with multiple drugs, with low compliance and a heavy disease burden. Most patients with hypertension are given oral antihypertensive drugs that may directly interact with microorganisms in the gastrointestinal tract. To avoid the influence of drug factors, previous researchers have often excluded patients receiving antihypertensive treatment. A cohort study examined 1003 patients who did not report antihypertensive medication use and suggested that the gut microbiota and host plasma metabolites were associated with BP in Chinese adults [15]. Another study showed that antihypertensive drugs were significantly associated with changes in gut microbiota [16]. Angiotensin receptor blockers (ARBs) are widely used as the first‐line antihypertensive drugs that can improve the performance of the gastrointestinal tract in hypertensive patients [17]. Some ARBs, such as valsartan, can significantly alter the gut microbes in animals [18]. We previously demonstrated that angiotensin‐converting enzyme inhibitor or ARB administration in hypertensive patients results in significant differences in intestinal flora composition and serum metabolites between those with WC and poorly controlled BP [19]. However, little is known about whether intestinal flora is involved in the antihypertensive effect of ARB, and the exact role and effect of gut microbiota after ARB administration remain unclear.

In this work, we performed fecal microbiota transplantation (FMT) in ARB‐treated animals and WC hypertensive patients to spontaneously hypertensive rats (SHRs), assessed the influence of ARB‐modified gut microbiome on BP, vasculature, and intestine, and then evaluated the alterations in intestinal transcriptome and serum metabolome, and explored the critical bacteria conferring the antihypertensive potential of ARB.

## RESULTS

### ARB‐modulated gut microbes ameliorate high BP and vascular damage

**Figure 1:**
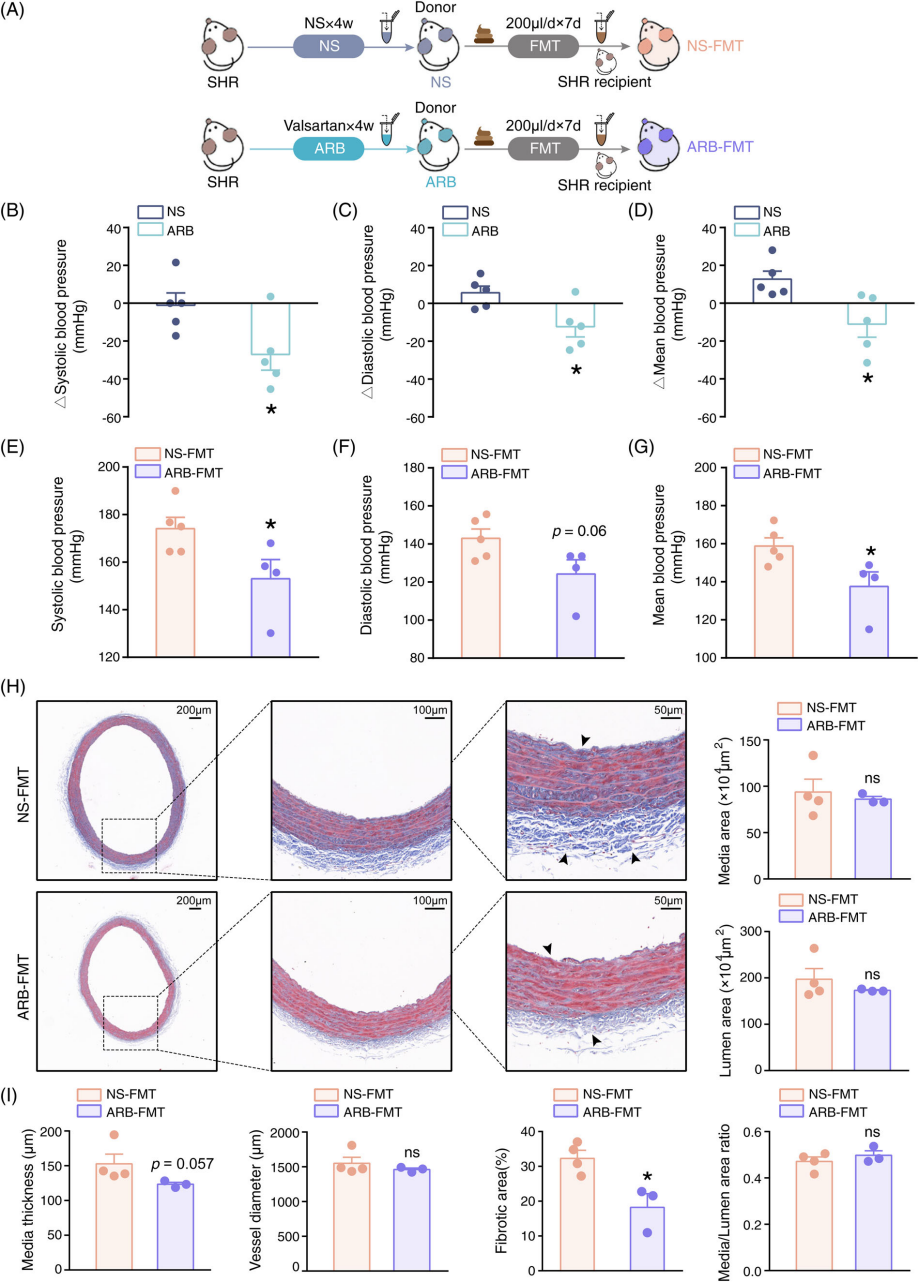
Intestinal microbiota following valsartan administration decreased blood pressure and alleviated vascular fibrosis in SHR recipients. (A) Timeline for the studies in NS or valsartan‐treated donor animals and subsequent FMT to SHR recipients. (B–D) Shifts of systolic, diastolic, and mean blood pressure in donor SHRs before and after NS or valsartan (ARB) (7.4 mg/kg/day) treatment. *n* = 5 per group. (E–G) Systolic, diastolic, and mean blood pressure (BP) of recipient naive SHRs post FMT. *n* = 5 for NS‐FMT, *n* = 4 for ARB‐FMT. (H) Representative photomicrographs and magnifications of Masson staining with aortic cross‐sections in SHRs receiving NS or valsartan‐modified gut microbiota. Scale bars are 200, 100, and 50 μm, respectively. (I) Quantified media thickness, vessel diameter, fibrotic area, media area, lumen area, and media/lumen area ratio of aortas in each FMT recipient group. *n* = 4 for NS‐FMT, *n* = 3 for ARB‐FMT. Data are presented as mean ± SEM. *p < 0.05; ns: not significant. ARB, angiotensin receptor blockers (valsartan); ARB‐FMT, rats inoculated with fecal microbiota from valsartan treated SHR; FMT, fecal microbiota transplantation; NS, normal saline; NS‐FMT, rats receiving gut microbiota from normal saline‐treated SHR; SHR, spontaneously hypertensive rat.

To assess whether the antihypertensive effects of ARB could be improved through the intestinal flora, we conducted FMT in donor SHRs who received valsartan therapy or not. SHRs treated with normal saline (NS) were considered negative control donors. The experimental design and protocol for FMT in recipients are shown in Figure 1A. As expected, valsartan administration effectively decreased the systolic, diastolic, and mean BP (MBP) of the SHRs (Figure 1B–D). However, in hypertensive animals that received FMT from ARB donors, BP dramatically improved, except for diastolic BP (DBP), compared with negative control donors (Figure 1E–G). In addition, to explore whether ARB could maintain their antihypertensive effect in the absence of gut microbiota, antibiotics were utilized before ARB administration to deplete the bacteria. Pretreatment with antibiotics was performed following the protocol described in the previous study [5]. Our findings revealed that treatment with ARB had no impact on SBP, DBP, and MBP levels in animals when antibiotics were applied (Figure S1A–C), indicating the impaired antihypertensive effect of ARB in the absence of gut microbiota.

As vascular remodeling has been previously regarded as the fundamental basis for hypertensive disease [20], we subsequently examined the impairment of the vasculature in FMT recipients. Morphological analyses of arteries revealed that the media thickness and area, vessel diameter, and lumen area, together with the media/lumen ratio representing vascular remodeling, were similar between the groups. In contrast, the severity of vascular fibrosis was significantly alleviated upon ARB‐FMT compared with NS‐FMT (Figure 1H,I).

Next, we determined the oxidative stress and production of reactive oxygen species (ROS) within the arteries in ARB‐FMT‐treated hypertensive rats. ARB‐FMT exerted antioxidative effects in aorta of hypertensive rats, as indicated by decreased ROS production (Figure S2A,B). Therefore, ARB treatment was thought to partly improve hypertensive vascular fibrosis damage and oxidative stress in a gut microbiome‐dependent manner. ARB reshaped gut microbiota, which suggested that it could exert a beneficial influence on hypertensive rats, with a partial extent similar to that observed in ARB animals.

### FMT from ARB‐rats rebuilds gut microbiota and affects intestinal gene profiles in SHRs

To confirm that the intestinal flora in SHR recipients was reconstructed after FMT, fecal microbiota analysis of NS‐FMT and ARB‐FMT rats was performed by sequencing the 16S rRNA gene. Taxonomic annotation revealed the dominant microbial phyla, classes, orders, families, and genera in each group (Figure S3A). Firmicutes and Bacteroidetes were the most abundant phyla observed in the SHRs that received the ARB‐modified microbiota. Classes of *Clostridia*, *Bacteroidia*, and *Erysipelotrichi*, and orders including *Clostridiales*, *Bacteroidales*, *Erysipelotrichales*, and families from *Ruminococcaceae*, *Lachnospiraceae*, and *Erysipelotrichaceae* were also the primary taxa observed in SHRs receiving ARB‐modified microbiota. At the genus level, we detected protective bacteria, such as *Blautia*, *Allobaculum*, and *Lactobacillus*, in a large proportion of SHR after ARB‐FMT. A total of 23 genera were identified with consistent directional changes when a comparison was made between the ARB and NS groups and between ARB‐FMT and NS‐FMT rats, including deficient *Lactobacillus* and *Desulfovibrio* in ARB and ARB‐FMT groups (Figure S4A).

Based on the taxonomic profiles identified at the operational taxonomic unit (OTU), alpha‐diversity analysis of Chao1, Shannon, Pielou, Simpson, Faith's phylogenetic diversity (Faith's pd), good coverage, and observed OTUs were performed to assess the diversity of the microbial community (Figure S3B). Among these indices, there were three major ecological parameters, including Chao richness, which is an estimate of the total number of OTUs present in the given community; Pielou evenness, which shows how evenly the individuals in the community are distributed over different OTUs; and Shannon diversity, which is the combined parameter of richness and evenness [21]. None of the examined indices significantly differed in ARB‐modified fecal microbiota‐treated rats (Figure S3B). Although stable performance in alpha‐diversity was observed after ARB‐FMT, the beta‐diversity analysis revealed distinct community structures in bacterial characteristics, showing significant differences between fecal samples from the NS‐FMT and ARB‐FMT groups (Figure S3C). Both principal coordinate analysis (PCoA) and nonmetric multidimensional scaling (NMDS) clustering analysis, which were frequently examined previously [22], derived from Bray Curtis, Jaccard, unweighted UniFrac, and weighted UniFrac distances, clearly separated the samples in the ARB‐FMT group from those in the other groups (Figure S3C). The beta‐diversity distances: Bray Curtis, unweighted UniFrac, and weighted UniFrac distances, have been widely used by researchers to assess differences in bacteria structure across samples [23].

For each distance, including Bray Curtis, Jaccard, unweighted UniFrac, and weighted Unifrac, the Unweighted pair‐group method with arithmetic means (UPGMA) hierarchical clustering tree further validated the similarity among NS‐FMT and ARB‐FMT samples, with pronounced separation detected using Jaccard and weighted Unifrac distance (Figure S5A–D). To test the statistical significance of beta diversity, permanova, anosim, and adonis analyses were conducted, emphasizing the dramatic differences in the microbial community structures of ARB‐FMT compared with NS‐FMT (Figure S5D).

**Figure 2:**
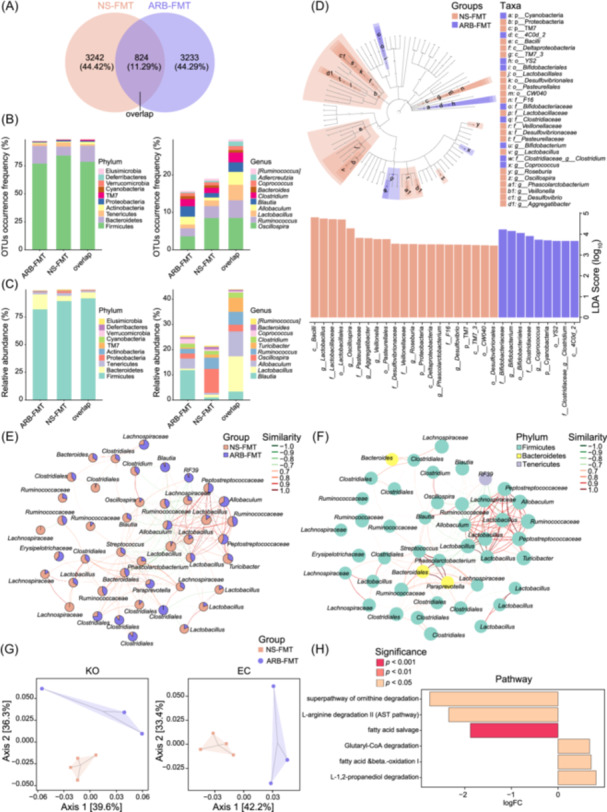
Gut bacteria and potential microbial functions discriminative between saline‐ and valsartan‐microbiota transplanted recipient rats. (A) Venn diagram describing the unique and shared number of operational taxonomic units (OTUs) detected in NS‐FMT and ARB‐FMT groups. The overlap denotes the shared OTUs in both groups. (B) Occurrence frequency of OTUs shared or unique between NS‐FMT and ARB‐FMT groups at phylum and genus levels. (C) Relative abundance of the top 10 most dominant phyla and genera overlapped between groups and those specified in each group. (D) Discrepant taxonomy constitution between SHRs receiving saline or valsartan‐treated microbiota is revealed with Linear discriminant analysis (LDA) effect size (LEfSe). The cladogram and bar plot of the LDA score depict significantly differentially enriched (DE) taxonomic compositions in different groups. The statistical significance of different taxa is defined by LDA scores (log_10_) > 2 and *p* < 0.05. (E) Network analysis based on the relative abundance of the top 50 dominant OTUs identifying the co‐occurrence or co‐exclusion relationships across gut microbial members. A node represents OTU, and the relative abundance ratio of OTU in different groups is shown in the pie chart. Different nodes, that is, OTUs, may belong to the same phylum or genus; therefore, there might be distinct nodes with the same name. SparCC algorithm was used to calculate the correlation matrix, and igraph package in the R software was used to construct the co‐occurrence correlation network. (F) OTUs in the co‐occurrence network were annotated to different phyla. The connection edge indicates the correlation between nodes. The red line indicates a positive correlation, and the green line indicates a negative correlation. Similarity, *r* value of correlation. SparCC algorithm was used to calculate the correlation matrix, and igraph package in the R software was used to construct the co‐occurrence correlation network. (G) Principal coordinate analysis plots based on the Bray Curtis distance of microbial functions in KEGG Orthology (KO) in the left panel (axis 1 and axis 2 explain 39.6% and 36.3% of the overall variation, respectively) and Enzyme Commission (EC) in the right panel (axis 1 and axis 2 explain 42.2% and 33.4% of the overall variation, respectively), respectively, as predicted by Phylogenetic Investigation of Communities by Reconstruction of Unobserved States (PICRUSt2). (H) Functional pathways are significantly distinct between groups based on the MetaCyc database. logFC, log_2_ (Fold Change of ARB‐FMT/NS‐FMT). *p* values are denoted in colors.

Moreover, the gut microbes responsible for the variations after ARB‐reshaped microbiota transplantation were elucidated. The shared and specific OTUs in each group are shown in the Venn diagrams (Figure 2A). Among the 7299 identified OTUs, 44.42% and 44.29% were detected uniquely in NS‐FMT and ARB‐FMT groups, respectively (Figure 2A). In addition, 824 OTUs were simultaneously observed in both groups. Detailed gut taxonomic information and microbial composition were described. At the phylum level, the specific or shared gut bacteria dominant in groups were Firmicutes, Bacteroidetes, and Tenericutes, either based on OTUs occurrence frequency or relative abundance (Figure 2B,C). For genera, the majority of exclusive or common OTUs between NS‐FMTs and ARB‐FMTs were annotated to *Oscillospira*, *Ruminococcus*, *Lactobacillus*, *Allobaculum*, *Blautia*, and *Clostridium* (Figure 2B). The abundance distribution of the communal genera across groups was quite different from that of the unique genera in each group (Figure 2C).

Taxonomic biomarkers with significant abundance differences in the NS‐FMT and ARB‐FMT groups were demonstrated by Linear discriminant analysis (LDA) Effect Size (LEfSe) analysis (Figure 2D). The cladogram illustrates the bacterial markers of each group in the taxonomic hierarchy. Bacteria significantly enriched in each group are shown according to the distribution of LDA values. *Bifidobacterium*, *Coprococcus*, and *Clostridium* were prominently elevated in SHRs receiving ARB‐modified microbiota, while *Lactobacillus*, *Oscillospira*, *Aggregatibacter*, *Veillonella*, *Roseburia*, *Phascolarctobacterium*, and *Desulfovibrio* were notably reduced (Figure 2D). Co‐abundance networks revealed complicated connections, primarily among *Phascolarctobacterium*, *Streptococcus*, *Paraprevotella*, *Peptostreptococcaceae*, *Allobaculum*, and *Lactobacillus* (Figure 2E,F). Thus, these intestinal bacteria might be positively or negatively linked with each other and show symbiotic or competitive relationships upon ARB‐FMT treatment. Based on the potential functional prediction of Kyoto Encyclopedia of Genes and Genomes (KEGG) orthology (KO) and Enzyme Commission (EC) identified with Phylogenetic Investigation of Communities by Reconstruction of Unobserved States (PICRUSt2), scatter plots of PCoA distinguished the samples in ARB‐FMT from NS‐FMT into distinct clusters (Figure 2G). In addition, the capacities of the gut microbiota following ARB‐FMT were significantly enhanced in pathways of Glutaryl‐CoA degradation, fatty acid beta‐oxidation, and L‐1,2‐propanediol degradation but decreased in l‐arginine degradation, fatty acid salvage, and ornithine degradation (Figure 2H).

Given the vital role of compromised gut pathological changes in hypertension development [24], we next investigated whether relieved intestinal impairment was implicated in the BP‐lowering effect of the ARB‐modified microbiota. However, comparable results were observed when measuring villus length, tunica muscularis layer thickness, goblet cell amounts, and fibrotic area in the small intestine of the SHRs following FMT from NS or ARB donors (Figure S6A–D). Meanwhile, the positive staining of tight junction proteins, including claudin, occludin, and TJP1 within the small intestine was similar between the groups (Figure S7A–C). Although antihypertensive treatment with ARB showed beneficial effects on attenuating BP through the gut microbiota, it seemed to fail to exert profound protection on gut pathology in SHRs.

**Figure 3:**
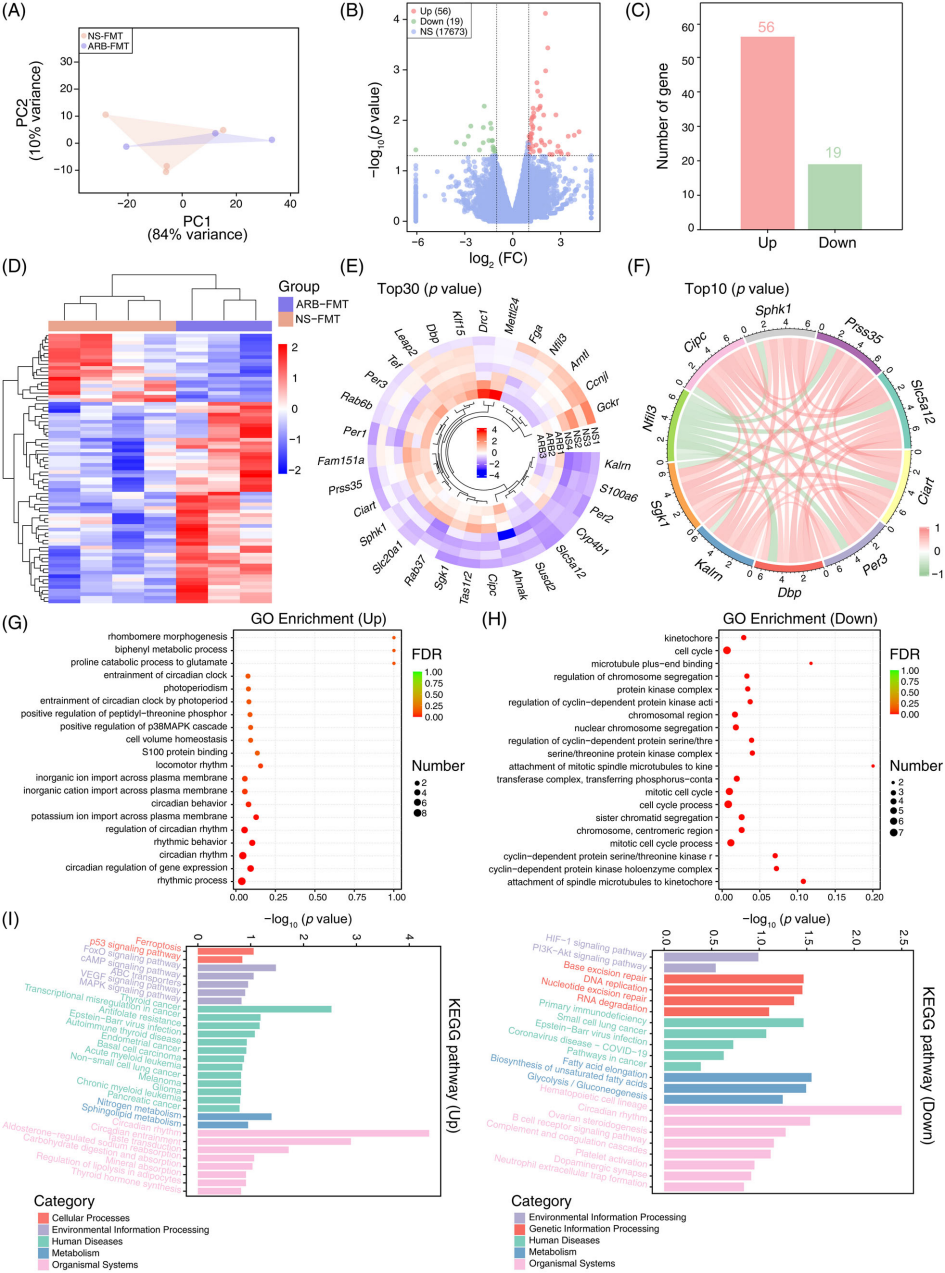
Transcriptome and RNA expression profiles within the intestine of valsartan microbiota‐treated SHRs. (A) Principal component analysis (PCA) based on RNA expression levels in intestinal samples was used to assess differences between groups. PC1 could explain 84% of the variance between groups, and PC2 accounts for 10%. (B, C) Volcano plots and bar plots describe the distribution and number of altered genes in valsartan‐modified microbiota‐treated SHRs when compared with the NS‐FMT controls. Genes with *p* < 0.05 and |log_2_FC | >1 are regarded as significantly different; FC, Fold Change. Up in red shows the number of significantly enhanced genes in the ARB‐FMT group (*n* = 56); Down in green represents the number of dramatically reduced genes (*n* = 19); NS, the number of genes not significantly altered (*n* = 17,673). *n* = 4 for NS‐FMT, *n* = 3 for ARB‐FMT. (D) Heatmap illustrating the relative abundance of genes significantly discriminative between groups. The abundance of differentially enriched genes is transformed into Z scores by subtracting the average and dividing the standard deviation. The *Z* score is negative in blue when the abundance is less than the mean and is positive in red when the abundance is higher than the mean. (E) Tree plot depicting the relative abundance of the top 30 most significant genes disparately enriched in groups. (F) Correlation relationship across the top 10 differently expressed genes between groups. Red lines denote a positive association; green lines represent a negative association. (G, H) The top 20 most enriched (according to FDR, adjust *p* value) Gene Ontology (GO) categories of genes significantly augmented (Up) or dramatically depressed (Down) in the ARB‐FMT group. Number, the number of Up or Down genes annotated to each GO term. The X‐axis represents the rich factor, the differentially enriched gene number ratio versus total annotated gene number in each GO term. (I) The top 20 (according to *p* value) KEGG pathways of the genes enriched (Up) or deficient (Down) in ARB‐FMT potentially participated in cellular processing, environmental information processing, human diseases, metabolism, organismal systems, or genetic information processing. Distinct categories are depicted in colors.

Consequently, transcriptome alterations in host gut tissues were investigated to explore other mechanisms. According to the expression profiles of intestinal genes, we observed similar and undistinguished scatter in the different groups using principal component analysis (PCA) plots (Figure 3A). Differential analysis demonstrated that ARB‐FMT treatment significantly augmented the expression of 56 genes and abated the expression of 19 genes in the gut (Figure 3B–D). The most discrepant genes between groups included circadian‐associated repressor of transcription (*Ciart*), period circadian regulator 1 (*Per1*), period circadian regulator 2 (*Per2*), period circadian regulator 3 (*Per3*), CLOCK‐interacting pacemaker (*Cipc*), cytochrome P450 family 4 subfamily b polypeptide 1 (*Cyp4b1*), sphingosine kinase 1 (*Sphk1*), Kruppel‐like factor 15 (*Klf15*), nuclear factor interleukin 3 regulated (*Nfil3*), and aryl hydrocarbon receptor nuclear translocator‐like (*Arntl*) (Figure 3E). Among the top 10 altered genes, *Nfil3* was negatively associated with the others (Figure 3F). Interestingly, GO enrichment analysis indicated remarkably enhanced potentials of the rhythmic process, circadian regulation of gene expression, and circadian rhythm in ARB‐FMT rats but deficient abilities to constitute cyclin‐dependent protein serine/threonine kinase and protein kinase holoenzyme complex (Figure 3G,H). Meanwhile, pathway analysis based on the KEGG database confirmed a significant increase in the host genes functioning in the circadian rhythm, nitrogen metabolism, ferroptosis, and FoxO signaling pathways (Figure 3I). In addition, the suppressed genes mainly participated in the hematopoietic cell lineage, immunodeficiency, fatty acid elongation, and hypoxia‐inducible factor 1 (HIF‐1) signaling pathway (Figure 3I).

### Restructuring of the serum metabolome by ARB‐FMT into SHRs

The small‐molecule metabolites produced by intestinal flora can pass through the gut barrier, be transported into the systemic circulation, mediate the effect of the gut microbiome on the host, and play a critical role in hypertension. Therefore, we examined the serum metabolic characteristics of recipients following FMT in ARB‐treated rats. Hierarchical clusters showing similarity between samples revealed a close linkage in each group under ESI‐ and ESI+ modes (Figure S8A,E). Score plots of the multivariate statistical analysis, including PCA, partial least‐square discriminant analysis (PLS‐DA), and orthogonal partial least‐square discriminant analysis (OPLS‐DA), further demonstrated significant discrepancies in the serum metabolic features of the NS‐FMT and ARB‐FMT groups (Figure S8B,C,D,F,G,H). The relative serum levels of all detected metabolic compounds in ESI+ and ESI‐ are shown in Figure S9A,B. The statistical distribution of differently enriched compounds in the NS‐FMT and ARB‐FMT groups was identified using volcano plots (Figure S9C–F). Overall, 1700 and 474 metabolic features were prominently reduced in ARB‐FMT rats, as detected in ESI‐ and ESI+ modes, and 1218 compounds in ESI‐ together with 172 in ESI+ significantly increased. The serum levels of these metabolic biomarkers, distinct between the groups, are illustrated in heat maps (Figure S9G,H). The m/z distributions of all the varied metabolic compounds in the ARB‐FMT group are shown in Figure S9I,J.

Based on the m/z, these compounds were further matched with the standards in databases, including the Human Metabolome Database and Massbank, and annotation information was acquired. Among the annotated metabolites, 42 were found in the ARB‐FMT group, such as Pyrrole‐2‐carboxylic acid, 3‐(2‐Hydroxyphenyl)propanoic acid, N‐Acetylserotonin, 6beta‐Hydroxytestosterone, and Thromboxane B2 (Figure S10A–D). In addition, 13 identified metabolites were sharply elevated after ARB‐specific microbiota transplantation, as represented by oxoglutaric acid, 2‐Oxo‐4‐methylthiobutanoic acid, and Sphingosine 1‐phosphate (Figure S10A–D). The strong correlation across these statistically different enriched metabolites implied a complicated interaction within serum metabolic properties (Figure S10E). We found that most of the metabolites affected by ARB‐FMT participated in pathways associated with metabolism, such as bile secretion, unsaturated fatty acid biosynthesis, arginine metabolism, proline, alanine, aspartate, and glutamate (Figure S10F–H). Next, we calculated the direct connections between metabolites and potentially involved pathways. Oxoglutaric acid, l‐glutamic acid, and l‐aspartic acid were suggested to be the core metabolites in the network, showing high frequency and correlation with multiple pathways (Figure S10I). Taken together, microbial transplantation from ARB donors has potent effects on the serum metabolome.

### High BP levels in SHRs are partially restored by WC‐FMT

Our recent work profiled the gut microbiome and serum metabolome in hypertensive patients and elucidated the association between antihypertensive therapy and ARB [12]. Patients benefiting from ARB treatment and showing WC hypertension are believed to possess ameliorated dysbiosis and restored gut microbiota homeostasis, at least to some extent. Human donors were included in FMT studies to further confirm and validate the crucial role of intestinal flora upon ARB modification in the treatment of hypertension and gain a further understanding of the key bacteria that may serve as the link between ARB and host therapeutic effects.

**Figure 4:**
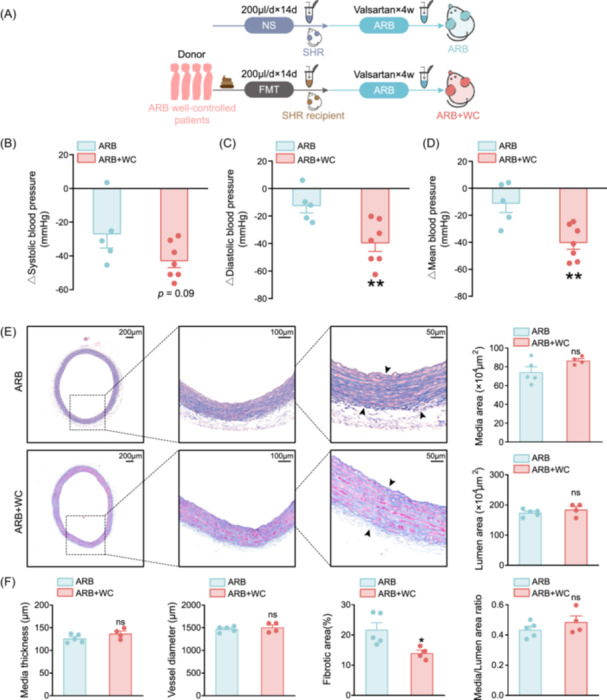
Fecal microbiota from hypertensive patients treated with ARB improved the therapeutic efficacy of valsartan in SHRs. (A) Study flow for SHRs administrated with valsartan and simultaneously received WC hypertensive patients intestinal flora transplantation or not. WC, hypertensive patients with WC hypertension under ARB therapy as donors for FMT. ARB + WC, SHR recipient animals with both FMT from WC and valsartan interventions. (B–D) Decrease in systolic, diastolic, and mean BP in SHRs following ARB or ARB + WC administration. The reduction of systolic (B), diastolic (C) mean BP (D) in the ARB + WC group was significantly higher than that in the ARB group. *n* = 5 for ARB, *n* = 7 for ARB + WC. (E) Representative images of Masson staining performed with aortic cross‐sections. Scale bars are 200, 100, and 50 μm, respectively. (F) Media thickness, vessel diameter, fibrotic area, media area, and lumen area, as well as media/lumen area ratio of the aorta, are quantified. Data are presented as mean ± SEM. *n* = 5 for ARB, *n* = 4 for ARB + WC *p < 0.05; **p < 0.01, ns, not significant. ARB, angiotensin receptor blockers; BP, blood pressure; FMT, fecal microbiota transplantation; SHR, spontaneously hypertensive rat; WC, well‐controlled.

Experiments were performed to investigate whether the BP of SHRs would be further alleviated after treatment with WC‐FMT. Surprisingly, neither SBP, DBP, nor MBP in SHRs was significantly improved by WC‐FMT (Figure S11A–C); thus, we speculated that gut microbes from WC patients alone might not be sufficient enough to reduce BP in SHR but could enhance the therapeutic effects of ARB. Hence, ARB was administrated following WC‐FMT treatment to explore whether pretreatment and the presence of partly restored gut microbiota from WC hypertensive donors would prominently facilitate the protective effect of subsequent ARB therapy. We addressed the efficacy of gut microbiota from WC hypertensive patients in altering the effects of ARB on BP and hypertensive damages (Figure 4A). SHRs who underwent FMT from WC donors before ARB therapy and those without FMT served as controls. Unsurprisingly, the gut microbiota from WC hypertensive donors enhanced the BP‐lowering effect of valsartan (Figure 4B–D). Again, microbial influence on arteries was mainly detected in alleviating fibrosis but was not evident in the vascular media and lumen (Figure 4E,F). Moreover, oxidative stress in the vasculature was found to be comparable between animals subjected to FMT (Figure S12A). Our results showed that the protective effect of ARB against the development of hypertension was partially due to gut microbes. It is speculated that there might be crucial bacteria as potential mediators of BP regulation and antihypertensive therapeutic effects in SHRs receiving microbiota from patients with WC hypertension.

### Manipulation of the gut microbiota and transcriptome by supplementing bacteria from WC donors

Hence, the intestinal flora that characterizes FMT versus non‐FMT SHRs under ARB administration was determined. Similar to the observations in NS‐FMT and ARB‐FMT rats, phyla including Firmicutes and Bacteroidetes, classes of *Bacilli* and *Clostridia*, orders of *Lactobacillales* and *Clostridiales*, families of *Lachnospiraceae*, and *Ruminococcaceae* were dominant in the animals (Figure S13A). *Blautia*, *Ruminococcus*, and *Lactobacillus* were the most abundant genera in all groups. Surprisingly, the alpha diversity of fecal microbial communities, as indicated by Chao1, Shannon, Pielou, Simpson, Faith's pd, and observed species, was considerably elevated after FMT, whereas the coverage was depressed (Figure S13B). Abnormally reduced microbial richness and evenness have been recognized as hallmarks of gut microbiota dysbiosis in hypertension [4, 21], and the shifts in alpha diversity elicited by FMT have been suggested as an essential protective aspect. Additionally, Bray Curtis, Jaccard, unweighted UniFrac, and weighted UniFrac dissimilarity distances among samples revealed significantly different community structures in the ARB and ARB + WC groups, with clearly segregated clusters in the PCoA and NMDS plots (Figure S13C). Likewise, UPGMA identified distinctions between groups, except for the weighted Unifrac distance (Figure S14A–D). In addition, the statistical significance of the beta diversity discrepancy was confirmed with permanova, anosim, and adonis (Figure S13D).

**Figure 5:**
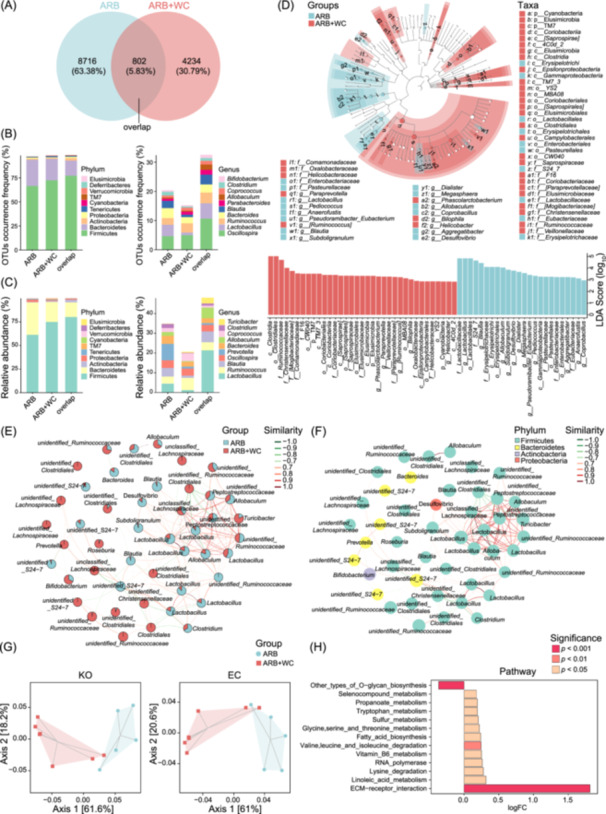
Heterogeneity of intestinal microbial composition and functional capacity between valsartan‐treated SHRs subjected to WC microbiota. (A) Venn diagram showing the distribution of specific and overlapped OTUs between groups. (B) The occurrence frequency of OTUs is simultaneously or uniquely detected in groups illustrated at the phylum and genus levels. (C) Relative abundance of the top 10 most enriched phyla and genera detected in groups. (D) LEfSe with cladogram and bar plot of LDA score uncovers the discrepancy in gut microbial composition between SHRs receiving merely valsartan or valsartan together with FMT. LDA scores (log_10_) > 2 and *p* < 0.05 represent statistical significance, and significantly, DE taxa are colored according to groups. (E, F) Network analysis of the top 50 most abundant gut microbial members. Nodes represent OTUs, which are displayed according to the relative abundance ratio in distinct groups (E), or the corresponding phyla assigned to (F). The connection line represents the correlation between OTUs. Red, a positive correlation; green, a negative correlation. Similarity, *r* value of correlation. (G) Bray Curtis distance of microbial functions in KO and EC as predicted by PICRUSt2 is shown in the PCoA plot. (H) MetaCyc pathways prominently different between groups. logFC, log_2_ (Fold Change of ARB + WC/ARB). ARB, angiotensin receptor blockers; BP, blood pressure; FMT, fecal microbiota transplantation; OTU, operational taxonomic unit; PCoA, principal coordinate analysis; SHR, spontaneously hypertensive rat; WC, well‐controlled.

A total of 802 OTUs were detected to coexist in ARB and ARB + WC rats, which mainly consisted of phyla Bacteroidetes and Firmicutes, and genera *Bacteroides*, *Ruminococcus*, and *Oscillospira* (Figure 5A–C). Microbes markedly enriched in FMT plus ARB‐treated animals were *Phascolarctobacterium*, *Paraprevotella*, *Bilophila*, and *Helicobacter*, whereas the depleted bacteria included *Pediococcus*, *Megasphaera*, *Dialister*, *Aggregatibacter*, *Coprobacillus*, *Lactobacillus, Desulfovibrio*, and so forth (Figure 5D). *Lactobacillus*, *Allobaculum*, and *Peptostreptococcaceae* showed complex interactions (Figure 5D). Moreover, the microbial function capacity, as indicated by the Bray Curtis distance based on KO and EC, was further affected by WC‐FMT, which promoted the metabolism of linoleic acid and tryptophan and the degradation of valine, leucine, and isoleucine, but suppressed O‐glycan biosynthesis (Figure 5G,H).

**Figure 6:**
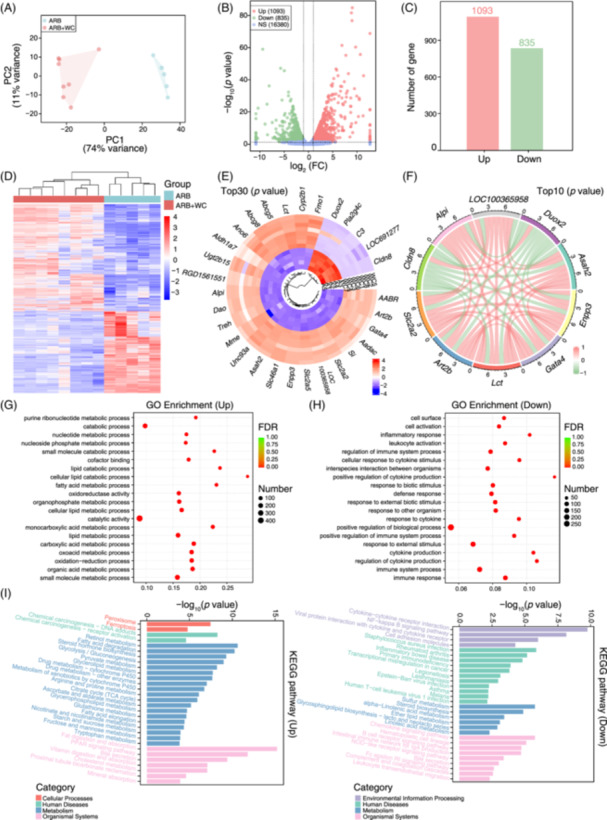
Transcriptome profiles of RNA in the intestine are affected by WC microbiota. (A) PCA based on RNA expression profiles in intestinal samples. PC1 accounts for 74% of the variance between groups, and PC2 explains 11%. (B, C) Distribution and number of varied genes in WC microbiota‐treated SHRs when compared with the ARB group. Genes with *p* < 0.05 and |log_2_FC | >1 are significantly different; FC, Fold Change. Up/Down, enhanced (*n* = 1093)/reduced (*n* = 835) in ARB + WC; NS, not significantly altered (*n* = 16,380). *n* = 5 for ARB, *n* = 8 for ARB + WC. (D) Relative abundance of genes significantly dissimilarly expressed between groups. (E) Tree plot shows the relative abundance of the groups' top 30 most significantly varied genes. (F) Correlation relationship among the top 10 DE genes. Red lines indicate a positive association; green lines indicate a negative association. (G, H) The top 20 most significantly enriched GO terms annotated by genes significantly increased (Up) or dramatically suppressed (Down) in ARB + WC. Dots are sized by the number of Up or Down genes within each term. The *x*‐axis denotes the ratio of DE gene number versus total annotated gene number in each term. (I) The top 20 most prominent KEGG pathways annotated by genes enriched (Up) or decreased (Down) in ARB + WC. ARB, angiotensin receptor blockers; PCA, principal component analysis; SHR, spontaneously hypertensive rat; WC, well‐controlled.

Comparison of intestinal injuries between the groups showed improved pathological changes after FMT (Figure S15A–D). The villus length and goblet cells were significantly elevated. In contrast, the thickness of the tunica muscularis and the area of fibrosis were reduced by the gut microbiota of WC donors (Figure S15A–D). Histopathological staining indicated no evident effect of WC‐FMT on tight junction proteins (Figure S16A–C). FMT also led to profound shifts in the gene expression profiles, as measured by the intestinal transcriptome (Figure 6). PCA showed disparate characteristics in the ARB + WC group relative to the controls without FMT (Figure 6A). Moreover, 1093 dramatically increased, and 835 genes decreased (Figure 6B–D). Interestingly, multiple enzyme genes, such as *Asah2* (N‐acylsphingosine amidohydrolase 2), *Lct* (lactase), *Alpi* (alkaline phosphatase), *Si* (sucrase‐isomaltase), *Aadac* (arylacetamide deacetylase), *Dao* (d‐amino‐acid oxidase) and *Duox2* (dual oxidase 2), and so forth, were affected by FMT (Figure 6E). Among these, *Duox2* was negatively linked to other enzyme genes (Figure 6F). The genes enhanced by FMT were implicated in monocarboxylic acid, fatty acid metabolism, and lipid catabolism, while those depleted played a crucial role in the immune system and inflammatory response (Figure 6G,H). Based on KEGG pathway analysis, we further confirmed elevated potentials toward fatty acid degradation, tryptophan metabolism, and the PPAR signaling pathway in the intestine of the ARB + WC group and inhibited cytokine receptor interaction, chemokine signaling pathway, and intestinal immune network (Figure 6I).

### Circulating metabolites relevant to WC‐FMT

These findings indicated that the intestinal microbiota of WC patients undergoing ARB therapy strongly influenced gut functions in hypertensive animals. To expand on this idea and increase our understanding of its impact on microbially influenced metabolites, metabolites were assessed using an untargeted metabolomics approach. The data obtained under ESI‐ showed a noticeable difference in clustering ARB and ARB + WC samples, which was also validated under ESI+ (Figure S17A–H). The relative abundances of all detected compounds are shown in Figure S18A,B. In addition, we found a multitude of different metabolites between the groups when analyzing the serum metabolome, with 1961 plus 341 compounds inhibited and 2093 plus 764 promoted by WC‐FMT (Figure S18C–F). These altered compounds following FMT are described in terms of their relative levels and m/z (Figure S18G–J).

Further, we evaluated the metabolome alterations in response to FMT. There were 28 reduced and 64 elevated metabolites showing statistical variation, such as 3‐Hydroxyphenylacetic acid and linoleic acid, 6beta‐hydroxytestosterone, Thromboxane B2 and N‐Acetylserotonin, Chenodeoxycholic acid, and d‐synephrine (Figure S19A–D). Next, a correlation analysis was used to uncover the co‐abundance relationships (Figure S19E). KEGG pathway analysis demonstrated that the differentially enriched metabolites were involved in glycine, serine, threonine and phenylalanine metabolism, bile secretion, and primary bile acid biosynthesis (Figure S19F–H). The network linking metabolites to pathway items revealed increased l‐Carnitine, Chenodeoxycholic acid, and glycochenodeoxycholic acid, while depressed taurocholic acid was associated with bile acid biosynthesis and secretion (Figure S19I). Among the metabolites with a pivotal role in phenylalanine metabolism, p‐hydroxyphenylacetic acid, phenylacetic acid, and 3‐(2‐Hydroxyphenyl) propanoic acid were enhanced, whereas 3‐Hydroxyphenylacetic acid was decreased (Figure S19I). Overall, the metabolic status of animals was impacted to a great degree by FMT administration.

### Correlation among the hypertension phenotype, microorganisms, metabolites, and functional genes

The Spearman test was used to analyze the correlation among the discrepant microorganisms, serum metabolites, intestinal genes between groups, and hypertension phenotype. In the SHRs receiving saline and valsartan‐treated microbiota, various distinct gut microbes between groups such as *Clostridium*, *Veillonella*, *Bifidobacterium*, *Aggregatibacter*, and *Lactobacillus* were significantly associated with the circulating metabolites, including 13‐l‐Hydroperoxylinoleic acid, Chenodeoxycholic acid, and Eicosadienoic acid, which subsequently linked to BP levels of the host (Figure S20A, Table S1). Furthermore, multiple intestinal flora were related to the transcriptome profiles, which were further associated with the hypertension phenotype (Figure S20B, Table S2). For instance, *Lactobacillus* was positively correlated with genes such as *Fga*, *Nfil3*, *Knstrn*, and so forth, which were also positively connected with SBP, DBP, or MBP. *Bifidobacterium* was positively associated with *Per2*, *Per3*, and *Rab6b*, which were negatively correlated with SBP, DBP, and MBP. In addition, the co‐occurrence network elucidated that most of the intestinal genes were highly relevant to crucial metabolites (Figure S20C). *Sphk1* and *Slc20a1* had a negative relationship with 13‐l‐Hydroperoxylinoleic acid, while *Ccdc34* and *Knstrn* were positively linked to Chenodeoxycholic acid. *Fga* showed a positive relationship with Eicosadienoic acid, whereas *Per2* and *Per3* showed inverse associations.

Meanwhile, the altered gut bacteria in response to WC‐FMT, including *Helicobacter*, *Blautia*, *Dialister*, *Megasphaera*, and *Phascolarctobacterium*, were dramatically associated with serum metabolites, such as 3‐Hydroxybutyric acid, 2‐Oxo‐4‐methylthiobutanoic acid, 3‐Hydroxyphenylacetic acid, and Phenylacetic acid. Moreover, these pivotal compounds were also correlated with hypertension phenotypes in the host (Figure S21A, Table S3). In addition, specific intestinal flora was linked to the transcriptome profiles associated with BP levels (Figure S21B, Table S4). Notably, *Blautia* was negatively correlated with genes such as *Ace*, *Apoa1*, and *Cyp17a1*, which were also negatively connected with DBP or MBP. *Megasphaera* was positively associated with *Tcfl5*, which in turn was positively correlated with MBP. The co‐abundance network illustrated intricate relationships between intestinal transcriptome profiles and serum metabolome (Figure S21C). Briefly, *Prodh1* showed a positive relationship with 3‐Hydroxybutyric acid, and *Galk1* showed a contrasting result. Moreover, *Lrrc8e* was negatively linked to Phenylacetic acid, while *Cyp2d4* showed an inverse association.

## DISCUSSION

There is a growing recognition of the complicated interactions between gut microbes and drugs and their potential contribution to the effectiveness of medical therapy against diseases [25, 26, 27]. For example, recent studies have shown that the microbiota‐derived metabolite indole‐3‐acetic acid has clinical implications for chemotherapy efficacy in pancreatic cancer [26]. In addition, the composition of intestinal microbes is believed to be a predictor of pharmacotherapeutic efficacy [28]. Moreover, the gut microbiome has been suggested to diminish the clinical efficacy of the inflammatory bowel disease drug 5‐aminosalicylic acid [29].

It has been reported that losartan‐mediated alterations in the gut microbiome exert beneficial effects on the vasculature and gut immune system in hypertensive animals [30]. In contrast, the gut microbiota might also reduce the efficacy of pharmacological interventions [31], leading to poorly controlled BP in hypertensive patients. In clinical practice, the variability in the efficacy of widely used antihypertensive drugs highlights the importance of microbiome‐based personalized medicine. We previously discovered distinct variations in the intestinal microbiota and metabolites corresponding to the outcome of angiotensin‐converting enzyme inhibitors and ARB treatment [19]; however, whether the gut microbiome in patients with WC hypertension contributes to its antihypertensive effect has not been systematically elucidated. Therefore, it is very important to identify the mechanisms through which intestinal flora improves hypertension in patients with WC.

Herein, we investigated changes in vasculatures and intestinal structures, fecal microbiota sequencing, intestinal transcriptome, and serum metabolomics data in SHRs subjected to FMT from WC patients and animals receiving ARB treatment. The underlying mechanism may be significant in elucidating the outcomes of BP control in clinical practice. Notably, the intestinal flora obtained from ARB‐treated SHRs was identified to have a prominent contribution to attenuation of BP, relieving vascular fibrosis and alleviating oxidative stress, which is consistent with previous reports using losartan [30]. Moreover, the finding indicated that gut microbiota can protect against vascular fibrosis and reactive oxygen accumulation and supports our observations [32, 33]. Next, we analyzed the functional consequences of fecal microbiota in WC patients who underwent ARB therapy and found that supplementation of microbiota from WC patients dramatically enhanced the efficacy of antihypertensive therapy toward rat models of hypertension when combined with ARB. Furthermore, an improvement in high BP, protection against vascular injuries, and restoration of pathological intestine abnormalities were observed in animals after WC‐FMT, similar to those detected in the ARB‐FMT group. The manifestations of hypertensive animals likely return to a configuration similar to that of normotensive controls, suggesting relative homeostasis driven by microbiome intervention.

Most importantly, we confirmed that the intestinal microbial community was restored in recipient animals after FMT treatment. Microbial analysis showed that the diversity, structural characteristics, composition, and functional potential of the gut flora were significantly modulated. Similarly, bacteria, including *Lactobacillus*, *Aggregatibacter*, and *Desulfovibrio*, were found to be simultaneously reduced by both animal‐ and human‐sourced FMT. The potential of *Lactobacillus* as an antihypertensive nonpharmacological strategy has been discussed in previous studies [34]. Simultaneously, investigators have recently clarified the enrichment of *Lactobacillus sp.* in elderly individuals with hypertension [35]. Moreover, *Lactobacillus*, an inflammation‐related bacterium, was found to be highly abundant in hypertensive patients with obstructive sleep apnea and inflammatory bowel disease [36, 37]. The positive association between *Lactobacillus* and trimethylamine N‐oxide, which promotes vasoconstriction and aggravates angiotensin II‐induced hypertension, implies a critical role of *Lactobacillus* in hypertensive disorders [38, 39]. In addition, studies have shown that hypertension is positively correlated with periodontopathic bacterial infections. Also, *Aggregatibacte* is a well‐known periodontal pathogen overrepresented in patients with hypertension or obstructive sleep apnea‐associated hypertension [40, 41]. In addition, the harmful genus *Desulfovibrio* in feces has been found to have a positive correlation with SBP and has been reported to increase in metabolic hypertensive animal models [42, 43]. Thus, we suggest that *Lactobacillus*, *Aggregatibacter*, and *Desulfovibrio* may be critical for determining or modulating the efficacy of antihypertensive therapy. Nevertheless, the mechanism through which these bacteria participate in BP control remains to be elucidated.

In recent research, *Megasphaera* was specifically dominant in poorly controlled hypertensive patients compared with untreated or WC individuals [19]. In addition, it has been elucidated that in hypertensive patients undergoing antihypertensive treatment, the abundance of *Megasphaera* was positively correlated with SBP [44], which aligns with our observation of *Megasphaera* suppression following FMT from WC patients. Meanwhile, *Bifidobacterium* was found to be enriched both in healthy controls and in patients receiving antihypertensive treatments but decreased in treatment‐naive patients [44], supporting the potential role of elevated *Bifidobacterium* detected in ARB‐FMT animals in the present study. In addition, *Clostridiaceae* and *Clostridium*, previously regarded as drastically increased bacteria in WC hypertensive patients [19], were found to be enhanced by ARB‐FMT in animals. Both *Clostridia* and *Clostridiales* were also prominent in the ARB + WC group. We further uncovered that *Bilophila* and *Paraprevotella*, which had lower abundance in the poorly controlled patients [19], proliferated following microbiota transplantation from WC hypertensive patients.

By investigating the serum metabolome, we considered that shifts in end‐products related to the gut microbiota may account for medical efficacy and mediate the function of FMT. We focused on significantly depressed 6beta‐Hydroxytestosterone and Thromboxane B2 levels because of their consistent alterations postmicrobiota transplantation. 6beta‐Hydroxytestosterone is a cytochrome P450 metabolite, and accumulating evidence shows that it causes vascular impairments in Ang II‐induced hypertension and cardiovascular pathophysiological changes [45, 46]. In fact, the intestine possesses the metabolic capacity to produce 6beta‐Hydroxytestosterone [47]. The arachidonic acid‐related metabolite Thromboxane B2 is a vasoconstrictor significantly elevated in the plasma and urine of patients with essential hypertension, which was confirmed to be involved in the development of hypertension in rats with chronic kidney disease rats [48, 49, 50]. Management with valsartan was associated with a decreasing trend in Thromboxane B2 [51]. Therefore, depleting 6beta‐Hydroxytestosterone and Thromboxane B2 in SHR recipients administered ARB‐modified microbiota might facilitate lower BP and suppress vascular damage.

Given the pivotal role of intestinal functions in BP modulation and hypertension development, transcriptome changes were determined by gene enrichment analysis. Herein, distinct gene profiles were observed in the intestinal walls after FMT, and our validation based on both animal‐ and human‐originated microbiota transplantation demonstrated profound variations. For instance, the reduction of *Nfil3* and *Arntl*, as well as the upregulation of *Ciart*, *Cipc*, *Per1*, *Per2*, *Per3*, *Tef*, serum/glucocorticoid regulated kinase 1 (*Sgk1*), d‐box binding PAR bZIP transcription factor (*Dbp*), pyruvate dehydrogenase kinase 4 (*Pdk4*), *Klf15*, and so forth, were consistently observed after FMT. Among these genes, *Nfil3*, *Arntl*, *Ciart*, *Cipc*, *Per1*, *Per2*, *Per3*, *Tef*, and *Dbp* are regulators of the circadian rhythm. *Nfil3* is a microbiota‐dependent regulator of mucosal homeostasis, whose rhythmic expression in the epithelium is influenced by compositional changes in commensal bacteria [52]. *Arntl* knockout has been suggested to substantially lower SBP in male animals [53], although another report indicated a significant negative correlation between *Arntl* gene expression and BP in hypertensive women [54]. In contrast, the *Per1* gene expression level in peripheral blood mononuclear cells was found to be negatively related to BP [54], thus being a potential protective factor. However, we found that mice lacking *Per1* showed significantly reduced BP [55], but *Per1*, *Per2*, and *Per3* gene levels tended to be lower in SHR adrenal glands than in controls [56]. Until recently, it was confirmed that the knockout of the circadian clock protein PER1 exacerbated the development of salt‐sensitive hypertension [57]. Furthermore, valsartan has been shown to restore the expression of circadian clock genes, including *Per1* and *Per2* [58]. The circadian rhythm is a sophisticated mechanism that affects the physiological activities of various molecules that influence metabolic pathways. A disrupted circadian rhythm can increase the risk of cardiovascular disease, hypertension, and metabolic syndrome. Based on our findings, ARB may modulate gut circadian rhythms in a microorganism‐dependent manner.

The downregulation of *Sgk1* in the colon of hypertensive patients was rescued by microbiota‐derived metabolites [59], which was also observed following FMT in our study. *PDK4* can potentially encode an enzyme that suppresses mitochondrial activity in favor of glycolysis [60]. Increased *PDK4* expression in lung pericytes has been linked to reduced endothelial–pericyte interactions and small vessel loss in pulmonary arterial hypertension [60]. As there might be a significant difference in the pathogenesis between systemic and pulmonary circulation, the role of *PDK4* in BP control remains to be explored. In addition, *KLF15* is an important negative regulator of cardiac hypertrophy [61, 62], which has been confirmed to be decreased in patients with hypertensive nephropathy [63]. Loss of *KLF15* was found to exacerbate pressure‐induced fibrosis injury [63]. Interestingly, some studies have reported that *KLF15* mitigates renal fibrosis and injury in hypertensive mice [64], which is in line with our observations in intestinal tissues.

We have comprehensively integrated the observations in the functional enrichment analysis of microorganisms, metabolites, and genes. It was noted that the microbial potentials in fatty acid oxidation and biosynthesis were enhanced in the ARB‐FMT or ARB+WC group, while the capacity in fatty acid salvage was suppressed. More intestinal genes were involved in the degradation of fatty acid in ARB+WC animals, and those genes reduced by ARB‐FMT played a role in fatty acid elongation. Despite these discordances, it was noteworthy that both intestinal transcriptome genes and serum metabolites in ARB‐FMT animals showed impaired function in unsaturated fatty acids biosynthesis. Meanwhile, the microbial pathway in lysine degradation was boosted following WC‐FMT, coinciding with increased metabolites that functioned in lysine degradation in the ARB‐FMT group. Our results support a recent study indicating that fatty acid biosynthesis, metabolism, and lysine degradation may have potential relevance to hypertension and antihypertensive drug response [65]. Moreover, we identified elevated functional enrichment of gut microorganisms in tryptophan and vitamin metabolism in the ARB+WC group, which resonated with similar findings for the intestinal genes. The gut microbiota is well known as a modifier of tryptophan metabolism, and there is increasing evidence suggesting a contributory role of microbiota‐derived tryptophan metabolites in BP regulation and hypertension [66]. Impaired tryptophan metabolic pathway in pregnancy has been implicated in the developmental programming of hypertension [67]. In addition, citrate is known as one of the TCA cycle intermediates, and impaired TCA cycle has been demonstrated to be related to high BP through energy balance and ion exchange [68]. Intriguingly, our findings uncovered enhanced capacities in the citrate cycle (TCA cycle) and mineral absorption of the intestinal genes in response to WC‐FMT administration, which was consistent with the functional alterations of serum metabolites. Notably, microbial gene function in the ABC transport system was significantly decreased in hypertensive patients and might be essential for healthy individuals [4]. In the current work, both prominent metabolites in the ARB+WC group and intestinal genes that varied following ARB‐FMT exhibited increased capacity in ABC transporters.

## CONCLUSION

ARB‐modified gut microbiota exerts protective roles in vascular remodeling and injury, metabolic abnormality and intestinal dysfunctions, which cooperate to lower the BP of the host. Our observations provide insights into the cross‐talk between gut microorganisms and hypertensive damage improvement during ARB therapies, which advances our understanding of the mechanisms underlying the response of hypertensive patients to clinical therapies.

## METHODS

All detailed methods are available in the online‐only Supplementary information.

## AUTHOR CONTRIBUTIONS

Jing Li, Ying Dong, and Jiu‐Chang Zhong conceived and designed the study. Jing Li, Si‐Yuan Wang, and Kai‐Xin Yan contributed to the literature search, conducted experiments, and collected data. Pan Wang, Ying Dong, Jie Jiao, Yi‐Dan Wang, and Mu‐Lei Chen collected the clinical samples. Jing Li and Si‐Yuan Wang analyzed and interpreted the data. Jing Li and Ying Dong wrote and revised the manuscript. Jiu‐Chang Zhong and Ying Dong supervised all the processes in this study. All the authors have read and agreed to the published version of the manuscript.

## CONFLICT OF INTEREST STATEMENT

The authors declare no conflict of interest.

## ETHICS STATEMENT

The ethics application of animal study (No. AEEI‐2023‐231) was approved by the Research Ethics Committee of the Capital Medical University. The ethics application of human study (No. 2022‐ke‐43) was approved by the Research Ethics Committee of the Capital Medical University.

## Supporting information


**Table S1.** Interrelationship across the discrepant metabolites between SHRs receiving saline or valsartan‐treated microbiota, and hypertension phenotype (|r| > 0.5).
**Table S2.** Interrelationship across the discrepant genes between SHRs receiving saline or valsartan‐treated microbiota, and hypertension phenotype (|r| > 0.5).
**Table S3.** Association between the varied circulating metabolites and hypertension phenotype following WC‐FMT (|r| > 0.5).
**Table S4.** Association between the varied circulating genes and hypertension phenotype following WC‐FMT (|r| > 0.8).

**Figure S1.** Antibiotic pretreatment leads to impaired antihypertensive effects of ARB administration.
**Figure S2.** Vascular oxidative stress in SHR was improved by valsartan‐modulated intestinal flora.
**Figure S3.** Profiles of gut flora in SHR recipients transplanted with NS or valsartan‐modified microbiota.
**Figure S4.** The intestinal microbial shift at the genus level in recipient animals post‐transplantation is partly shared with the donors between NS and ARB groups.
**Figure S5.** Hierarchical clustering analysis to evaluate the microbial similarity of SHRs receiving NS‐ or valsartan‐ microbiota.
**Figure S6.** Intestinal pathology of SHRs remained stable upon FMT from valsartan‐treated rats.
**Figure S7.** Tight junction proteins in the intestine of fecal microbiota transplanted recipient rats.
**Figure S8.** Cluster and separation analysis to characterize the profiles of serum metabolome in NS‐FMT and ARB‐FMT.
**Figure S9.** Enrichment analysis of serum metabolites detected in NS and valsartan FMT rats.
**Figure S10.** Identification and functional annotation of serum metabolites significantly varied between NS‐FMT and ARB‐FMT.
**Figure S11.** Fecal microbiota from hypertensive patients benefiting from ARB treatment exerts inapparent improvement of BP in SHRs.
**Figure S12.** Oxidative stress in the vasculature of ARB‐treated SHRs is unaffected by gut microbiota from WC hypertensive patients.
**Figure S13.** Gut flora derived from WC hypertensive patients influence the fecal microbiome of valsartan‐treated SHRs.
**Figure S14.** Hierarchical cluster of SHRs treated with ARB or ARB+WC according to gut microbial profiles.
**Figure S15.** Intestinal pathological improvement in ARB‐SHRs administrated with WC donors FMT.
**Figure S16.** Impacts on tight junction proteins in the intestinal tissue by WC fecal microbiota.
**Figure S17.** Global characteristics of serum metabolome patterns in ARB treated SHRs with and without FMT.
**Figure S18.** Enrichment of serum metabolites detected in ARB and ARB+WC groups.
**Figure S19.** Identification and functional capacity of serum metabolites significantly affected by fecal microbiota from WC donors.
**Figure S20.** Correlation of the microorganisms, serum metabolites, and intestinal gene with hypertension phenotype in SHRs receiving valsartan‐microbiota transplantation.
**Figure S21.** Association across the gut microbes, circulating metabolites and intestinal transcriptome, with hypertension phenotype following WC‐FMT.

## Data Availability

The data sets supporting the results of this study have been deposited in the Metabolights repository (accession number MTBLS7703) and the National Center for Biotechnology Information under BioProject accession code PRJNA956681 (https://www.ncbi.nlm.nih.gov/sra/PRJNA956681). The data and scripts used are saved in GitHub, https://github.com/lijing11999/Role-of-ARB-modified-gut-microbiota1. Supplementary materials (methods, figures, tables, graphical abstract, slides, videos, Chinese translated version, and updated materials) may be found in the online DOI or iMeta Science http://www.imeta.science/.
