# Supplementary material for: Intestinal microbiota by angiotensin receptor blocker therapy exerts protective effects against hypertensive damages
Source: Imeta. 2024 Jul 18;3(4):e222. doi: 10.1002/imt2.222 (PMC11316932; doi:10.1002/imt2.222)
Supplement: Supplementary file 2 — Figure S1. Antibiotic pretreatment leads to impaired antihypertensive effects of ARB administration. Figure S2. Vascular oxidative stress in SHR was improved by valsartan‐modulated intestinal flora. Figure S3. Profiles of gut flora in SHR recipients transplanted with NS or valsartan‐modified microbiota. Figure S4. The intestinal microbial shift at the genus level in recipient animals post‐transplantation is partly shared with the donors between NS and ARB groups. Figure S5. Hierarchical clustering analysis to evaluate the microbial similarity of SHRs receiving NS‐ or valsartan‐ microbiota. Figure S6. Intestinal pathology of SHRs remained stable upon FMT from valsartan‐treated rats. Figure S7. Tight junction proteins in the intestine of fecal microbiota transplanted recipient rats. Figure S8. Cluster and separation analysis to characterize the profiles of serum metabolome in NS‐FMT and ARB‐FMT. Figure S9. Enrichment analysis of serum metabolites detected in NS and valsartan FMT rats. Figure S10. Identification and functional annotation of serum metabolites significantly varied between NS‐FMT and ARB‐FMT. Figure S11. Fecal microbiota from hypertensive patients benefiting from ARB treatment exerts inapparent improvement of BP in SHRs. Figure S12. Oxidative stress in the vasculature of ARB‐treated SHRs is unaffected by gut microbiota from WC hypertensive patients. Figure S13. Gut flora derived from WC hypertensive patients influence the fecal microbiome of valsartan‐treated SHRs. Figure S14. Hierarchical cluster of SHRs treated with ARB or ARB+WC according to gut microbial profiles. Figure S15. Intestinal pathological improvement in ARB‐SHRs administrated with WC donors FMT. Figure S16. Impacts on tight junction proteins in the intestinal tissue by WC fecal microbiota. Figure S17. Global characteristics of serum metabolome patterns in ARB treated SHRs with and without FMT. Figure S18. Enrichment of serum metabolites detected in ARB and ARB+WC groups. Figure S19. Identif [file IMT2-3-e222-s002.doc]

**Supporting information to:**

Intestinal microbiota by angiotensin receptor blocker therapy exerts protective effects against hypertensive damages

**Running title:** Role of ARB-modified gut microbiota

Jing Li^1,2#^, Si-Yuan Wang^1,2#^, Kai-Xin Yan^1,2^, Pan Wang^1,2^, Jie Jiao^1,2^, Yi-Dan Wang^1,2^, Mu-Lei Chen^1,2^, Ying Dong^1,2^,*, [Jiu-](https://microbiomejournal.biomedcentral.com/articles/10.1186/s40168-016-0222-x#auth-17)Chang Zhong^1,2^,*

^1^Heart Center and Beijing Key Laboratory of Hypertension, Beijing Chaoyang Hospital, Capital Medical University, Beijing 100020, China

^2^Department of Cardiology, Beijing Chaoyang Hospital, Capital Medical University, Beijing 100020, China

#These authors contributed equally: Jing Li, Si-Yuan Wang.

***Correspondence:** dongying91@foxmail.com (Ying Dong); jczhong@sina.com ([Jiu-](https://microbiomejournal.biomedcentral.com/articles/10.1186/s40168-016-0222-x#auth-17)Chang Zhong)

**METHODS**

Animals and experimental groups

Spontaneously hypertensive rats (SHRs) aged 12 weeks were purchased from Charles River Laboratories. All the animals were housed at a temperature of 22 ± 1 ºC, relative humidity of 50 ± 1%, and a light/dark cycle of 12/12 h; they were fed with sterile food and water *ad libitum*.

The animals were divided into five groups: (1) NS group: Treated with normal saline (NS) by oral gavage every day for four weeks. (2) ARB group: Received valsartan (Novartis Pharmaceutical Co., Ltd, Beijing, China) at 7.4 mg/kg/day by oral gavage every day for four weeks. (3) NS-FMT group: Underwent fecal microbiota transplantation (FMT) from NS donors. (4) ARB-FMT group: Underwent FMT from angiotensin receptor blocker (ARB) donors. (5) ARB+WC group: Underwent FMT from well-controlled (WC) hypertensive patients and was treated with valsartan therapy for four weeks by oral gavage.

Hypertensive patients

To further understand the effects of ARB treatment on humans, we conducted a parallel study involving hypertensive patients. Hypertensive patients treated with ARB for at least four weeks were enrolled at the Beijing Chaoyang Hospital. Blood pressure (BP) of each patient was measured in the sitting position by physicians using an electronic sphygmomanometer. The average of three measurements was used as the final BP data, ensuing systolic BP < 140 mmHg and diastolic BP < 90 mmHg, indicating WC hypertension under antihypertensive medicine treatment.

Exclusion criteria

Exclusion criteria included: (1) Individuals with diseases related to intestinal flora such as cancer, heart failure, renal failure, stroke, peripheral artery disease, and chronic inflammation. (2) Patients taking other medications such as statins, aspirin, metformin, and other antihypertensive drugs (e.g., calcium channel blockers, beta blockers), or those who had taken oral antibiotics or probiotics in the past two months.

Ethical review

The detailed information including background, research purpose, study design, and experimental methods were submitted during application process. The experimental protocol was approved (AEEI-2023-231) by the Animal Care and Use Committee of Capital Medical University Beijing Chaoyang Hospital, and animal experiments were performed in compliance with the guidelines of the Animal Ethics Committee of the University. The study protocol was approved (2022-ke-43) by the Medical Ethics Committee of Beijing Chaoyang Hospital. Written informed consent was obtained from all patients prior to enrollment. The study conformed to the Helsinki Declaration of 1975 (as revised in 2008) concerning Human and Animal Rights.

FMT

Hypertensive patients with WC hypertension under ARB therapy were considered human donors for FMT. In addition, SHRs that received NS or valsartan were used as animal donors. Briefly, 5 g fecal samples were freshly harvested from human and animal donors and resuspended in cold PBS. The mixture was vortexed and incubated on ice for 15 minutes. Following centrifugation at 4 ºC 1,000 rpm for 5 min twice, the supernatants were mixed with an equal volume 20% (w/v) sterile glycerin. Recipient rats were treated with 200 μl inoculum by oral gavage every two days seven times. The recipient rats in the different groups were kept in separate cages and fed sterile food and water for four weeks.

BP measurement

The noninvasive tail-cuff method using the BP-2010A system (Softron, Tokyo, Japan) was used to determine the rats' systolic, diastolic, and mean BP. The rats were wrapped and placed in an insulation tube with a temperature of 37°C. The dark environment facilitated quiet status, and the appropriate temperature promoted blood circulation. The measurement was automatically initiated by pressing the start button, and the tail pulse wave was accurately monitored using infrared sensing technology. Data were recorded using a physiological data acquisition and analysis system.

Histological staining

The aorta and small intestine tissues were fixed with 10% paraformaldehyde, embedded in paraffin, and sectioned. Sections were stained to assess the histological pathology. The tissue sections were stained with hematoxylin-eosin to assess the morphological structure, and Masson’s trichrome staining was performed to evaluate the collagen area and fibrosis severity. The arteries were incubated with dihydroethidium (DHE) and counterstained with DAPI to produce red fluorescence when oxidized to ethidium by reactive oxygen species (ROS). Stained images were acquired using a fluorescence microscope (Nikon). Measurements of media thickness, vessel diameter, fibrotic area, media area, lumen area, media/lumen area ratio, and ROS-positive staining area of vasculatures, as well as villi lengths, tunica muscularis, percentage of goblet cells, and fibrotic area of intestines were performed using Image-Pro Plus software.

16S rRNA amplicon sequencing

Total DNA was extracted from stool samples. The quality of DNA was detected by 1.2% agarose gel electrophoresis, and quantification was performed using Nanodrop. A barcode sequence was added, and the variable region of the rRNA gene was amplified by PCR. Briefly, each sample was diluted to 20 ng/μl, and PCR reaction was performed with 5×reaction buffer 5 μl, 5×GC buffer 5 μl, dNTP (2.5 mM) 2 μl, forward primer (10 μM) 1 μl, reverse primer (10 μM) 1 μl, DNA template 2 μl, ddH_2_O 8.75 μl, Q5 DNA polymerase 0.25 μl. The primers utilized were as follows: forward, ACTCCTACGGGAGGCAGCA, and reverse, GGACTACHVGGGTWTCTAAT. The detailed condition of the amplified reaction was at initial denaturation 98 ºC 2 min, denaturation 98 ºC 15 s, annealing 55 ºC 30 s, extension 72 ºC 30 s, final extension 72 ºC 5 min, 10 ºC hold, and 25-30 cycles. The amplified products were quantified using a Quant-iT PicoGreen dsDNA Assay Kit on a microplate reader (BioTek, FLx800). The library was prepared with the Illumina TruSeq Nano DNA LT Library Prep Kit and purified by 2% agarose gel electrophoresis. The library was evaluated on an Agilent Bioanalyzer with Agilent High Sensitivity DNA Kit, quantified with Quant-iT PicoGreen dsDNA Assay Kit on Promega QuantiFluor system, denatured to a single strand, and subjected to paired-end sequencing on Illumina Novaseq-PE250 at Suzhou PANOMlX Biomedical Tech Co., LTD.

Fecal microbiota analysis

The raw high-throughput sequencing data underwent primer removal, mass filtering, denoising, and splicing using the DADA2 method in QIIME2 (2019.4). Classify-sklearn methods in QIIME2 (https://github.com/QIIME2/q2-feature-classifier) were used for taxonomic annotation in the Greengenes database (Release 13.8, http://greengenes.secondgenome.com/ comments). To assess the alpha diversity of the microbial community, richness was represented by chao1 and observed species index; diversity was indicated using shannon and simpson index; diversity based on evolution was described with Faith's PD index, evenness was represented by Pielou's evenness index, and Good's coverage index was used to depict coverage. Kruskal-Wallis rank sum test and Dunn’s test were used to determine the significance of differences across groups. Beta diversity distances such as Jaccard distance, Bray-Curtis distance, unweighted UniFrac distance, and weighted UniFrac distance were calculated to assess between-habitat diversity. Principal coordinate analysis (PCoA) and Nonmetric multidimensional scaling (NMDS) based on beta diversity distances were performed using the vegan and ggplot2 packages in R. The unweighted pair-group method with arithmetic means (UPGMA), a hierarchical clustering method to evaluate the similarity between samples, was conducted using the Stat and ape package in R. PERMANOVA, Anosim, and Adnois were calculated using QIIME2 to test the significance of the differences between groups. Clustering heatmaps were obtained using the pheatmap package in R. Linear discriminant analysis (LDA) Effect Size (LEfSe) analysis was a combination of nonparametric Kruskal-Wallis and Wilcoxon rank-sum tests and effect size in LDA, which was conducted on the Galaxy online analysis platform (http://huttenhower.sph.harvard.edu/galaxy/). Network analysis was performed using the igraph, ggraph, and RMThreshold packages in R based on SparCC methods. The potential functional capacity of gene sequences was predicted using Kyoto Encyclopedia of Genes and Genomes (KEGG) (<http://www.kegg.jp/>) and Phylogenetic Investigation of Communities by Reconstruction of Unobserved States (PICRUSt2). The MetagenomeSeq package in R was used to identify metabolic pathways with significant differences between groups.

Immunofluorescence staining

For immunofluorescence staining of claudin-, occludin-, and TJP1-positive cells in the intestine, paraformaldehyde-fixed tissues were embedded in paraffin and sectioned. The Sections were permeabilized with Triton X-100 and stained with antibodies specific to claudin, occludin, and TJP1 at 4 ºC overnight. The sections were then incubated with fluorescein isothiocyanate (FITC)-coupled secondary antibodies and counterstained with DAPI. Staining was examined using a fluorescence microscope (Nikon).

Intestinal RNA sequencing

Total RNA was extracted from colonic segments of the intestine. The concentration and purity were determined using an Agilent 2100 Bioanalyzer, and the polyA structure was used to purify mRNA. mRNA with polyA structure within total RNA was enriched by Oligo(dT) magnetic beads and fragmented to ~300 bp in length. The first strand of cDNA was synthesized using RNA as a template, and the second strand was synthesized using the first strand of cDNA as a template. Following library construction, PCR amplification was performed to enrich the library fragments, and 450 bp was used. The library was prepared with PCR using the NEBNext Ultra II RNA Library Prep Kit for Illumina kit (New England Biolabs Inc; Ipswich, Massachusetts, USA). According to the manufacturer’s instructions, the PCR reaction was performed with adaptor Ligated DNA 15 μl, NEBNext Ultra II Q5® Master Mix 25 μl, universal PCR Primer 5 μl, index (X) Primer 5 μl. The detailed condition of amplified reaction was at initial denaturation 98 ºC 30 s, denaturation 98 ºC 10 s, annealing 65 ºC 75 s, final extension 65 ºC 5 min, 4 ºC hold, and 8-13 Cycles. An Agilent 2100 Bioanalyzer was used to assess the library, and the effective concentration was examined. Libraries containing different index sequences were proportionally mixed. The mixed library was diluted to 2 nM, and a single-strand library was constructed. The prepared libraries were subjected to Paired-end Sequencing using the Illumina HiSeq sequencing platform.

Transcriptome analysis

The image files for sample sequencing were obtained and converted using a sequencing platform to generate raw data. Cutadapt was used to remove the sequence of 3' end-band connectors and reads with average quality scores lower than Q20 were also removed. The filtered high-quality sequences as clean data were blasted against the reference genome and the expression levels of each gene were calculated. HTSeq was used to analyze the read count and gene expression, and fragments per kilobase per million fragments (FPKM) were used for normalization. The DESeq package in R software was used to perform principal component analysis (PCA) according to the RNA expression profiles. For gene expression analysis, differentially expressed genes were those with |log_2_FoldChange| > 1 and a *p* < 0.05. Volcano plots and circle clustering maps depicting differentially expressed genes were obtained using the ggplots2 package and circlize package in R, respectively. Differentially expressed genes annotated by Gene Ontology (GO) terms or KEGG pathways were used to calculate the relative abundance for GO and KEGG analyses. The enrichment degree was measured using the rich factor, FDR value, and number of genes enriched in each term.

Liquid chromatography-tandem mass spectrometry (LC-MS/MS) measurement

Serum samples were mixed with 400 µL of methanol (Fisher Scientific, Loughborough, UK). The supernatants were concentrated and dried by centrifugation at 12,000 rpm at 4 ºC for 10 min. 2-chloro-l-phenylalanine solution prepared with 80% methanol was used to redissolve the sample. After filtering the supernatant with a 0.22 µm membrane, the samples were subjected to LC-MS/MS determination. Liquid chromatography was performed using a Vanquish UHPLC System (Thermo Fisher Scientific, USA) with ACQUITY UPLC ® HSS T3 (150×2.1 mm, 1.8 µm) (Waters, Milford, MA, USA). The column was maintained at 40 ºC, and the flow rate was 0.25 ml/min. For ESI+ mode, the mobile phases consisted of 0.1% formic acid in acetonitrile (A) and 0.1% formic acid in water. The separation was performed for 0–1 min, 2% A; 1–9 min, 2%–50% A; 9–12 min, 50%–98% A; 12~13.5 min, 98% A; 13.5~14 min, 98%–2% A; 14–20 min, 2% A. ESI- analysis was conducted with acetonitrile (B) and ammonium formate (5 mM). Separation was under 0~1 min, 2% B; 1~9 min, 2%~50% B; 9~12 min, 50%~98% B; 12~13.5 min, 98% B; 13.5~14 min, 98%~2% B; 14~17 min, 2% B. In addition, mass spectrometric measurements were performed using a Q Exactive (Thermo Fisher Scientific, USA) with an ESI ion source. The sheath gas pressure was 30 arb, gas flow at 10 arb, and spray voltage at 3.50 kV and -2.50 kV for ESI+ and ESI-, respectively. The capillary temperature was 325°C, MS1 m/z ranged from 100 to 1,000, and the resolving power was 70,000 FWHM. The MS/MS resolving power was 17,500 FWHM, and the normalized collision energy was 30 eV.

Metabonomics data processing and analysis

The raw data obtained were converted to mzXML using MSConvert in the ProteoWizard software package (v3.0.8789), and XCMS was applied for feature detection, retention time correction, and alignment. The MS/MS data were blasted with HMDB ([http://www.hmdb.ca](http://www.hmdb.ca/)), massbank (<http://www.massbank.jp/>), LipidMaps ([http://www.lipidmaps.org](http://www.lipidmaps.org/)), mzcloud ([https://www.mzcloud.org](https://www.mzcloud.org/)), and KEGG (<http://www.genome.jp/kegg/>), to identify metabolites by accuracy mass. The Robust LOESS signal correction (QC-RLSC) was used for data normalization and only ion peaks with relative standard deviations < 30% in QC were retained. Multivariate data analyses were conducted using Ropls software. Clustering models visualized as score plots were built using PCA, partial least-square discriminant analysis (PLS-DA), and orthogonal partial least-square discriminant analysis (OPLS-DA) according to metabolic profiles. OPLS-DA can discriminate metabolites between groups with variable importance on projection (VIP). *p* < 0.05 and VIP > 1 were considered statistically significant. Metabolites were subjected to pathway analysis using MetaboAnalyst, which facilitated the mapping of metabolites to KEGG pathways.

Statistical analysis

Statistical analyses were performed using the GraphPad software (version 9.0). Quantitative data are presented as mean ± SEM. Student’s *t*-test or Wilcoxon test was used for comparisons between independent groups, and one-way analysis ANOVA or Kruskal-Wallis tests were performed to compare multiple groups. *p* < 0.05 represented statistical significance.

**Figure Legends**

**
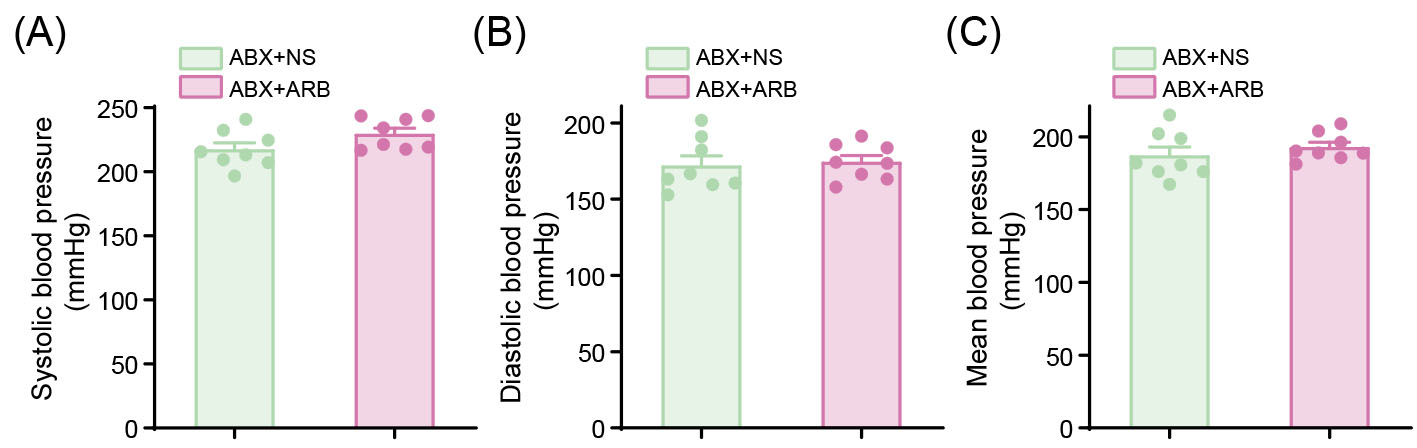
**

Figure S1. Antibiotic pretreatment leads to impaired antihypertensive effects of ARB administration. (A-C) SHRs were pretreated with antibiotics to examine whether ARB could achieve a profound antihypertensive effect in the absence of gut microbiota. Systolic, diastolic, and mean blood pressure in antibiotics-pretreated SHRs following NS or ARB administration were shown. *n* = 8/group. Data are presented as mean ± SEM. NS, normal saline; ARB, angiotensin receptor blockers (valsartan).

**
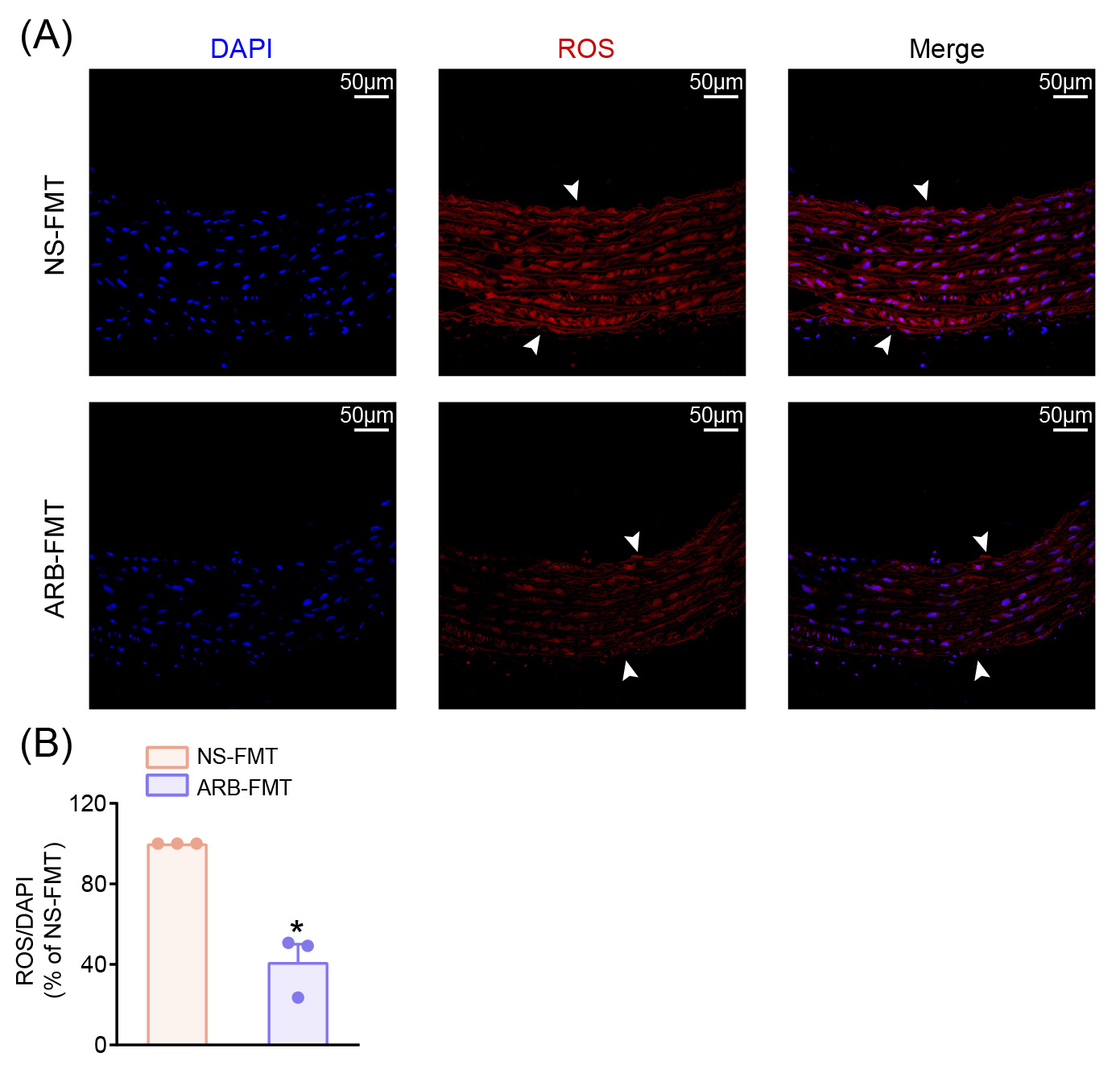
**

Figure S2. Vascular oxidative stress in SHR was improved by valsartan-modulated intestinal flora. (A) Arteries of SHRs following FMT were stained with dihydroethidium, and images of positive red staining for ROS were shown. Blue, nucleus dyed with DAPI; red, ROS stained with dihydroethidium. The white arrows indicate the ROS positive staining area. (B) Quantification of ROS positive fluorescence/nucleus positive fluorescence was normalized to the NS-FMT group. *n* = 3 per group. DAPI, 4',6-diamidino-2-phenylindole; ROS, reactive oxygen species. Data are expressed as mean ± SEM. *, *p* < 0.05.

**
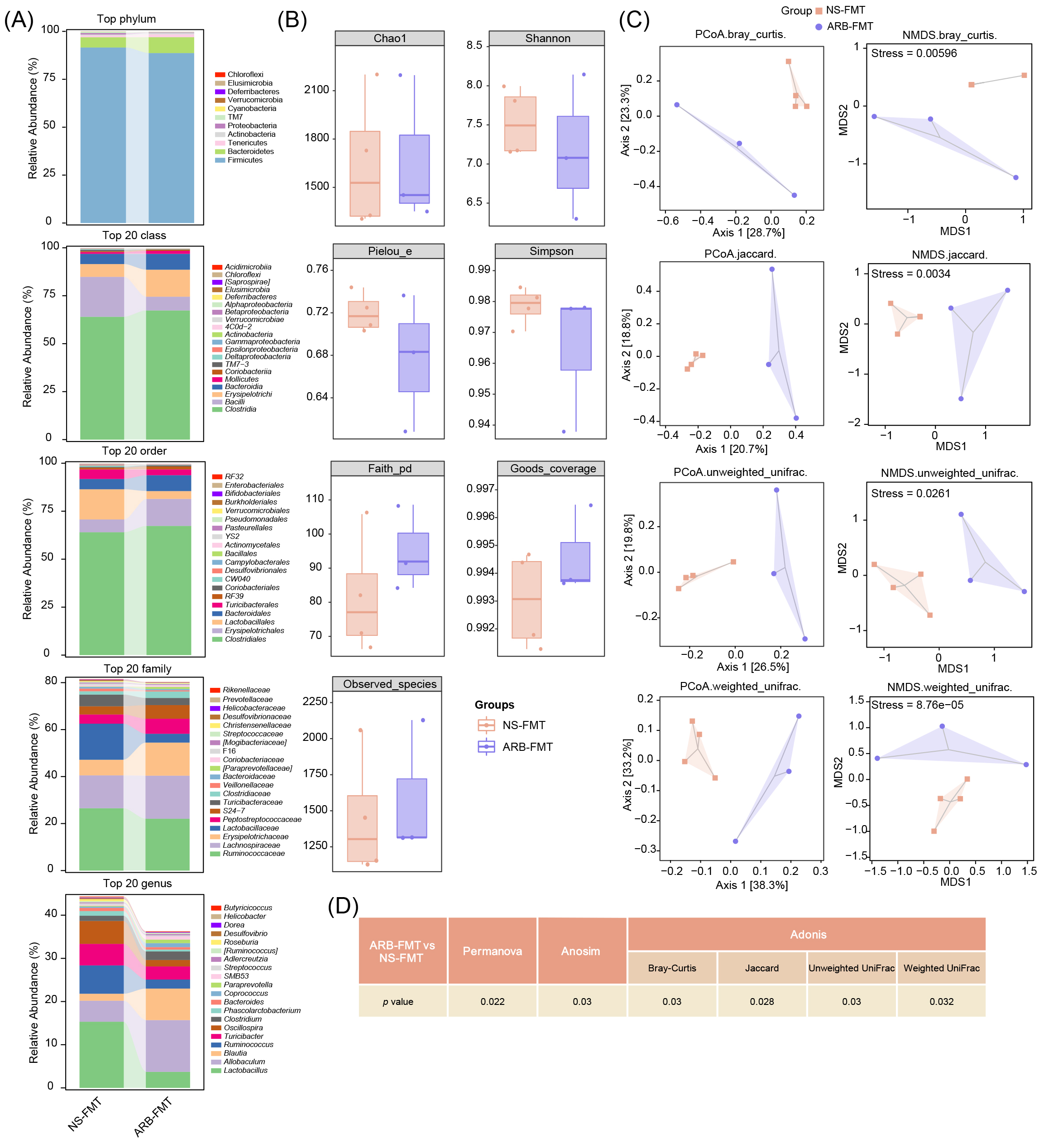
**

Figure S3. Profiles of gut flora in SHR recipients transplanted with NS or valsartan-modified microbiota. (A) Intestinal microbial composition in each group is illustrated with the relative abundance of the most abundant phyla, classes, orders, families, and genera, respectively. (B) Alpha diversity (within-habitat) parameters, including Chao1 richness, Shannon diversity, Pielou evenness, Simpson’s index, Faith’s phylogenetic diversity (Faith’s pd), Goods coverage, and Observed species are depicted. *n* = 4 for NS-FMT, *n* = 3 for ARB-FMT. Boxes represent the interquartile ranges, and lines inside denote medians. (C) Beta diversity (between-habitat), including PCoA and NMDS, is performed based on the Bray Curtis distance, Jaccard distance, unweighted Unifrac distance and weighted Unifrac distance, respectively. PCoA, principal coordinates analysis; NMDS, nonmetric multidimensional scaling. Axis 1 and 2 in PCoA plots indicate their extent to explain the group variations. The stress coefficient in NMDS plots is used to judge whether it can accurately reflect the distribution of data sorting. Stress < 0.2, certain explanatory significance; stress < 0.1, significant; stress < 0.05, representative. (D) *p* from permanova analysis, anosim analysis, and adonis analysis with Bray Curtis, Jaccard, unweighted Unifrac, and weighted Unifrac distance are applied to examine the significance of differences between groups.

**
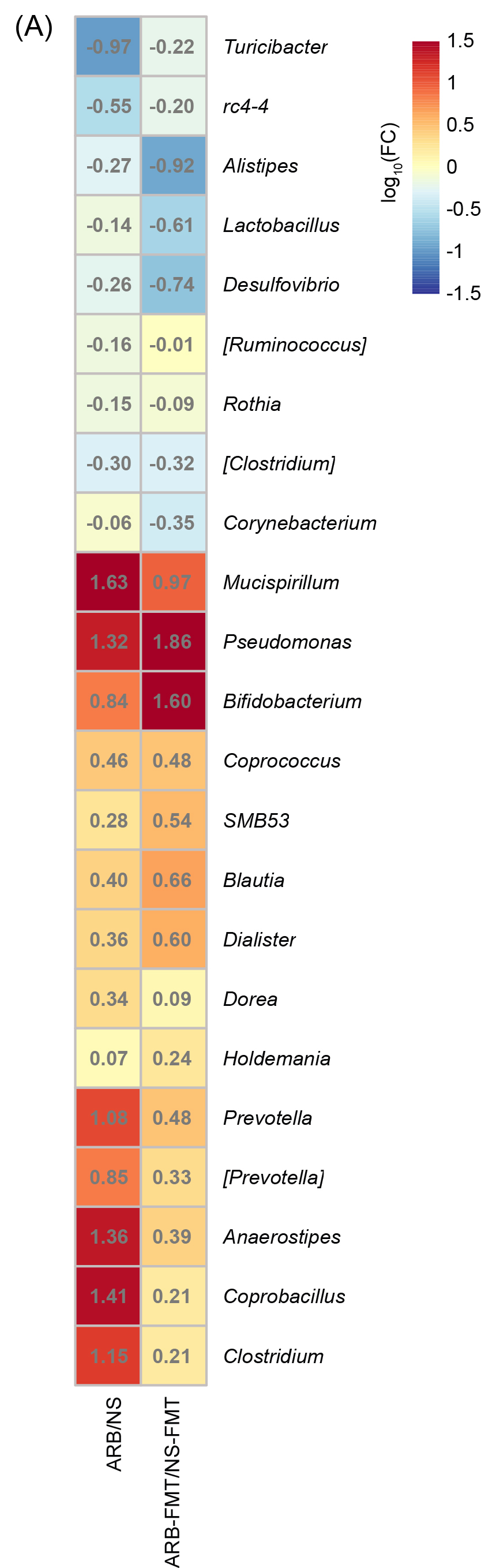
**

Figure S4. The intestinal microbial shift at the genus level in recipient animals post-transplantation is partly shared with the donors between NS and ARB groups. (A) The 23 genera exhibited consistent directional changes simultaneously when comparison was conducted between the ARB and NS group and between ARB-FMT and NS-FMT animals. A heat map comparing the abundance changes of these genera between groups was shown. Log_10_(FC) was labeled; FC, Fold Change. Red, more abundant in the ARB (vs. NS) and ARB-FMT (vs. NS-FMT) groups; blue, less abundant in the ARB (vs. NS) and ARB-FMT (vs. NS-FMT) group.

**
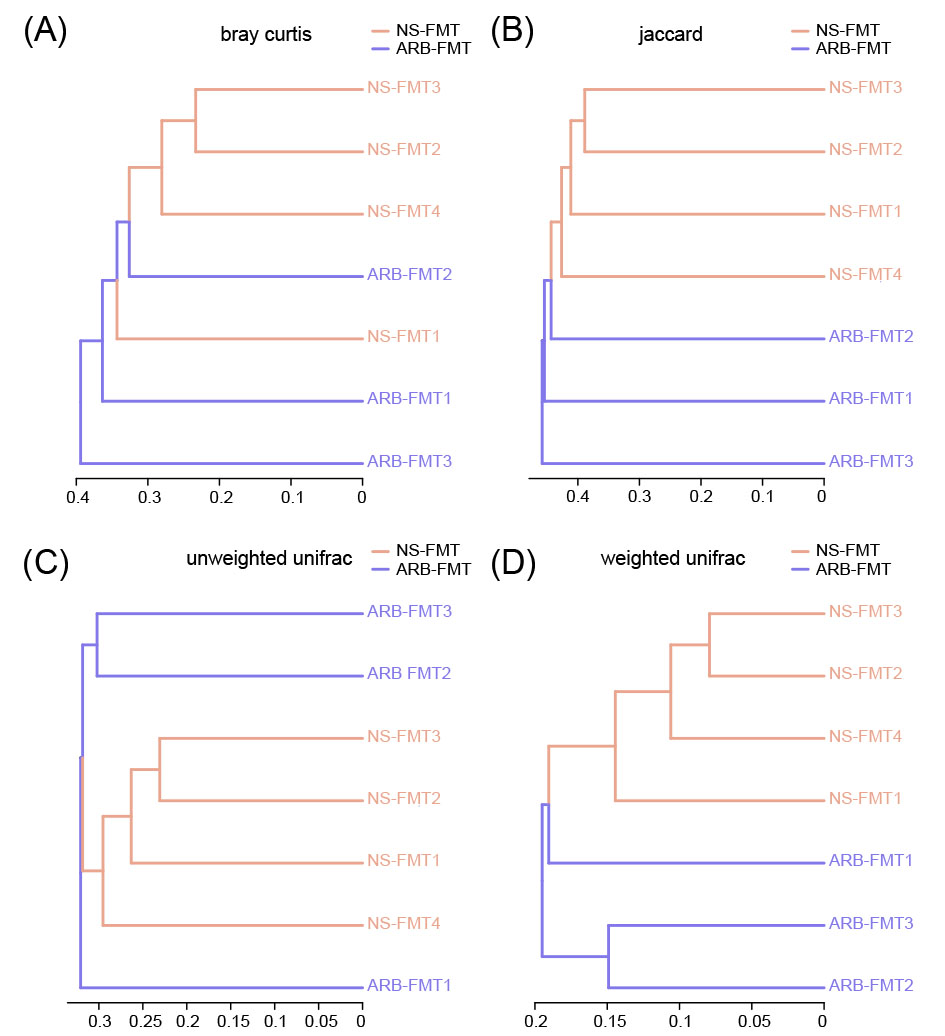
**

Figure S5. Hierarchical clustering analysis to evaluate the microbial similarity of SHRs receiving NS- or valsartan- microbiota. A-D, Cluster analyses with Bray Curtis (A), Jaccard (B), unweighted Unifrac (C), and weighted Unifrac (D) distance were conducted in individual samples based on the UPGMA method. The branch length of the clustering tree measures the effect of clustering.

**
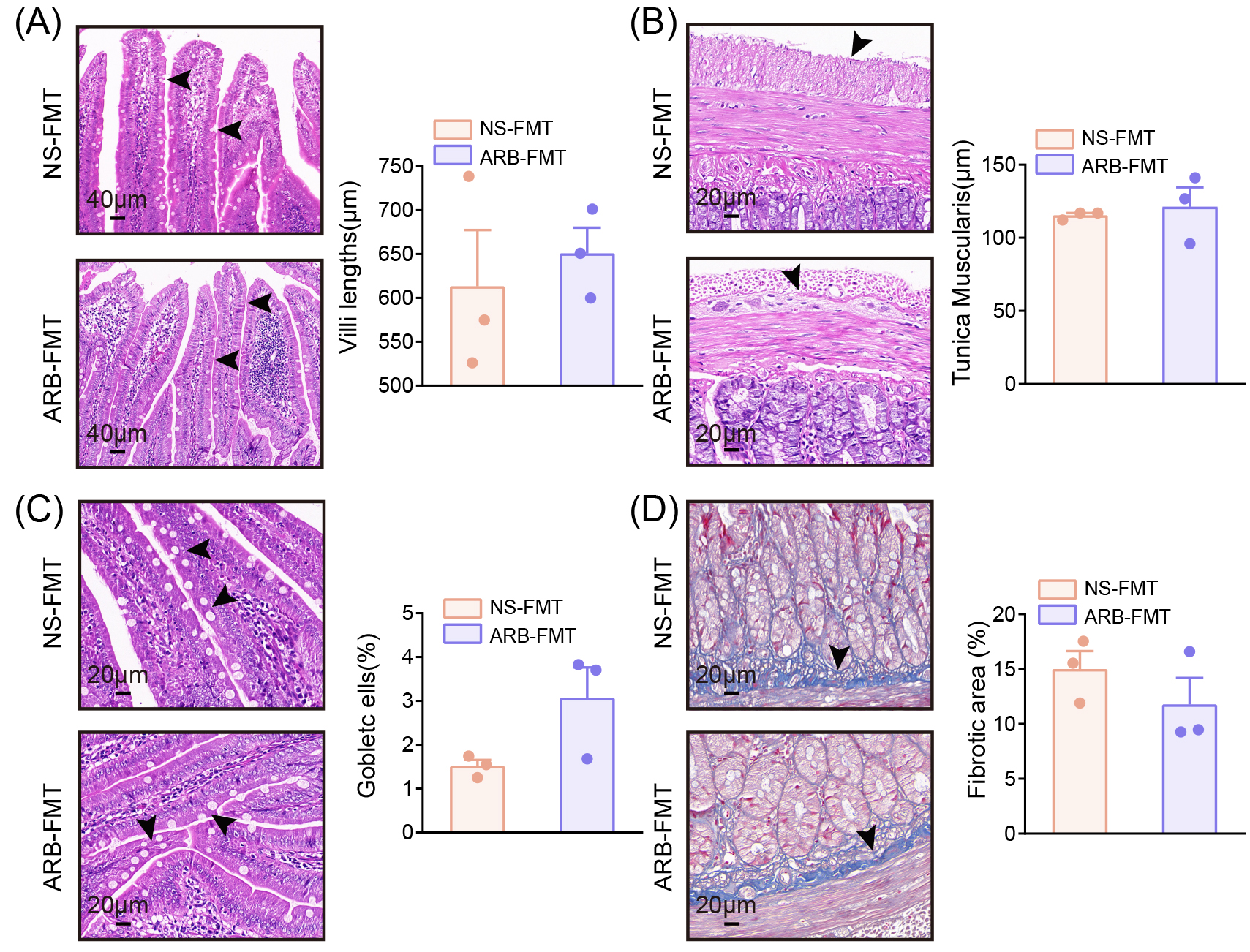
**

Figure S6. Intestinal pathology of SHRs remained stable upon FMT from valsartan-treated rats. (A) To measure the villi lengths, cross-sections of the intestine from SHR recipients are stained with hematoxylin and eosin. The arrows indicate villi. (B) The thickness of the tunica muscularis in recipient animals. The arrows denote tunica muscularis. (C) The percentage of goblet cells among epithelial cells in each group. The arrows indicate goblet cells. (D) To quantify the fibrotic area, the intestine of recipient SHRs was stained with Masson trichrome. The arrows point to fibrotic area. *n* = 3 per group. Data are presented as mean ± SEM. Scale bar: 20 μm.

**
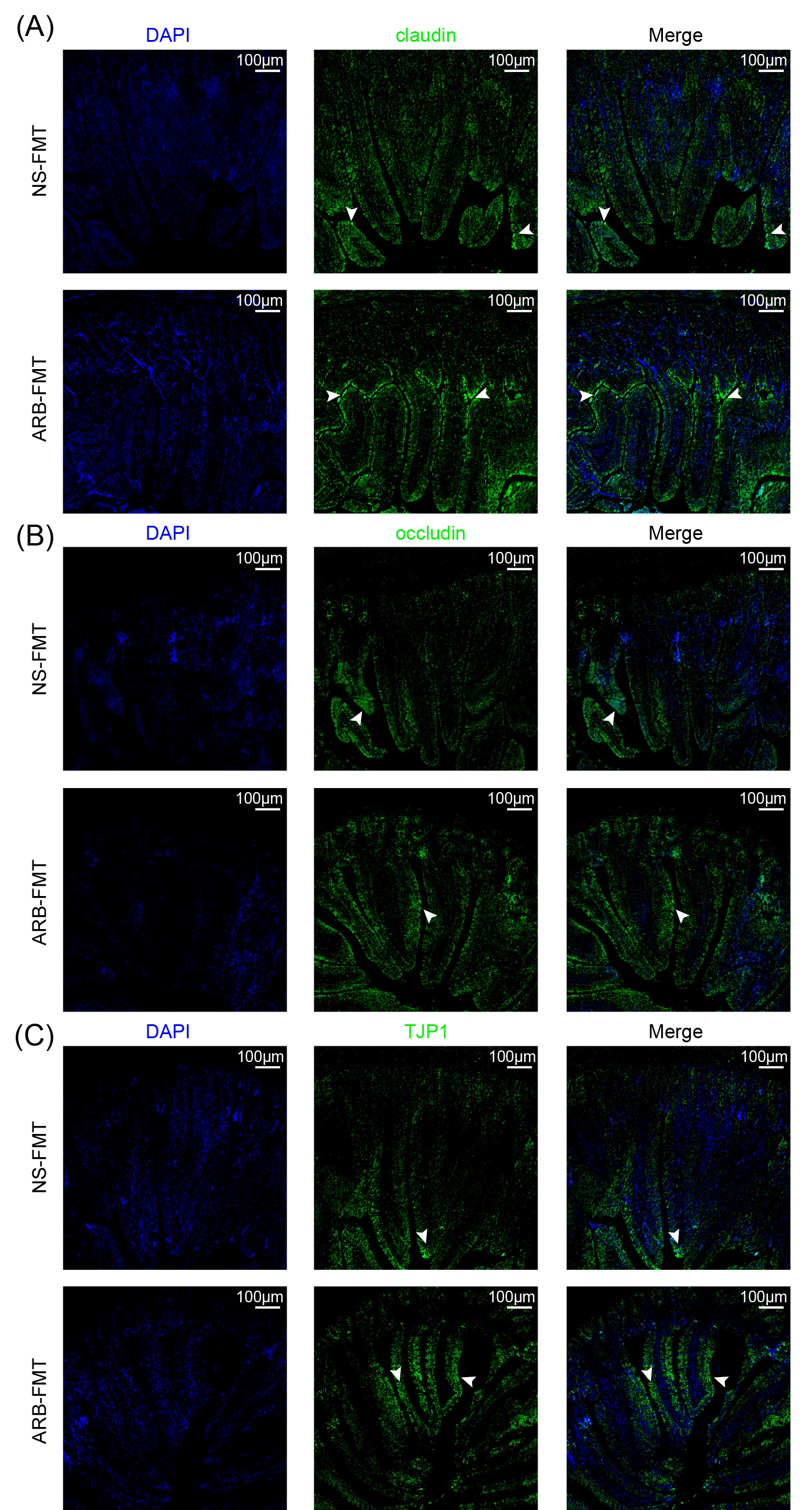
**

Figure S7. Tight junction proteins in the intestine of fecal microbiota transplanted recipient rats. (A-C) Representative immunofluorescence images assessing claudin, occludin, and TJP-1 in the intestinal tissue of SHRs administrated with normal saline or valsartan-modified microbiota. Positive stainings of claudin, occludin and TJP-1 are in green; nuclei are stained with 4',6-diamidino-2-phenylindole (DAPI, blue). The white arrows point to the immunofluorescence positive staining area. Scale bars are 100 μm.

**
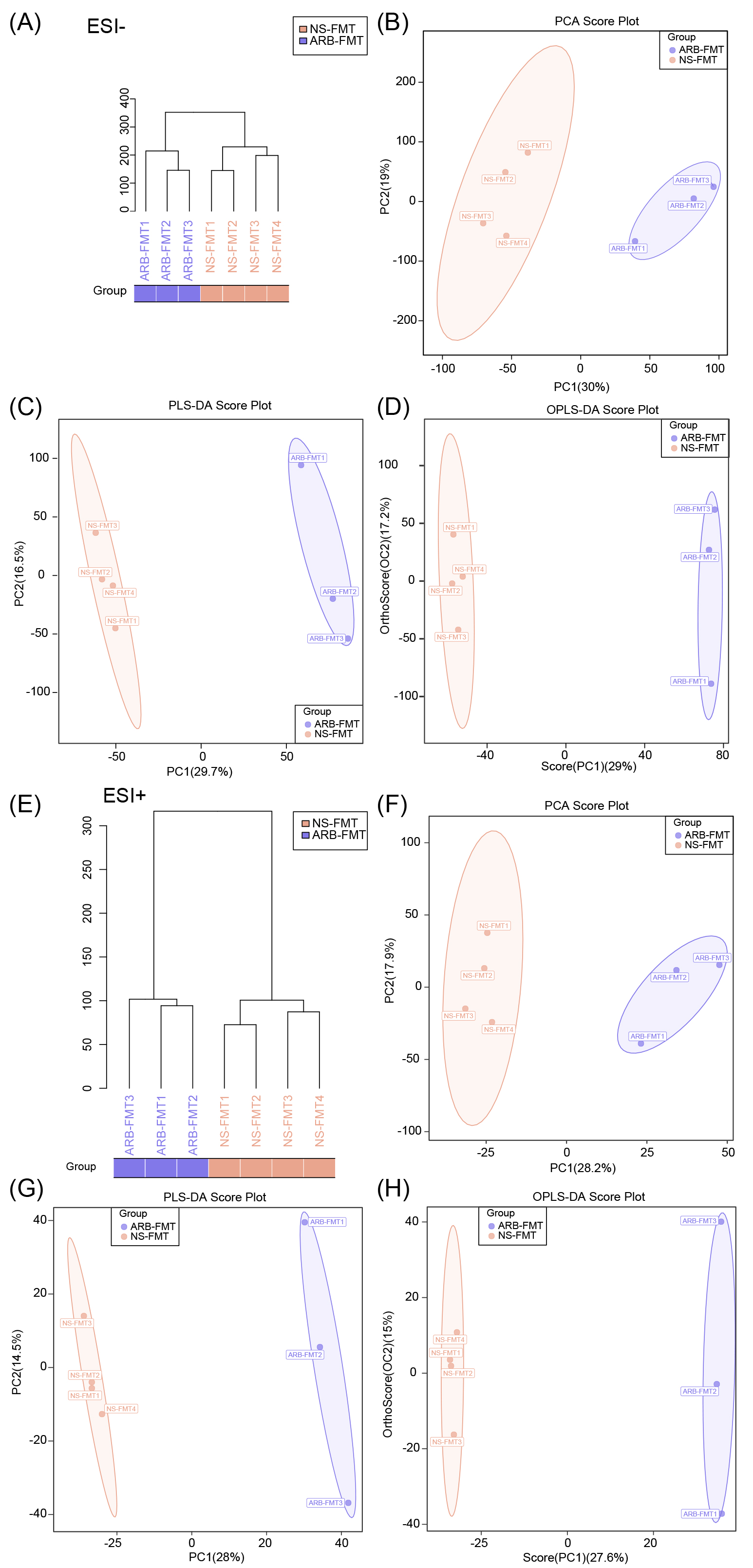
**

Figure S8. Cluster and separation analysis to characterize the profiles of serum metabolome in NS-FMT and ARB-FMT. (A) Hierarchical clustering tree for the metabolic profiles in ESI-mode of serum samples from NS-FMT and ARB-FMT groups is shown to examine the similarity between samples. The y axis represents the Euclidean clustering distance. (B-D) Principal Component Analysis (PCA), Partial Least Squares-Discriminant Analysis (PLS-DA), and Orthogonal Partial Least Squares Discriminant Analysis (OPLS-DA) identified in ESI- mode illustrates the metabolic discrimination and separation among samples. Each component denotes the corresponding explanation degree. (E) Hierarchical clustering analysis of samples according to the metabolic characteristics detected in ESI+ mode. (F-H) the performance of between-group differences in ESI+ mode was determined with PCA, PLS-DA, and OPLS-DA.

**
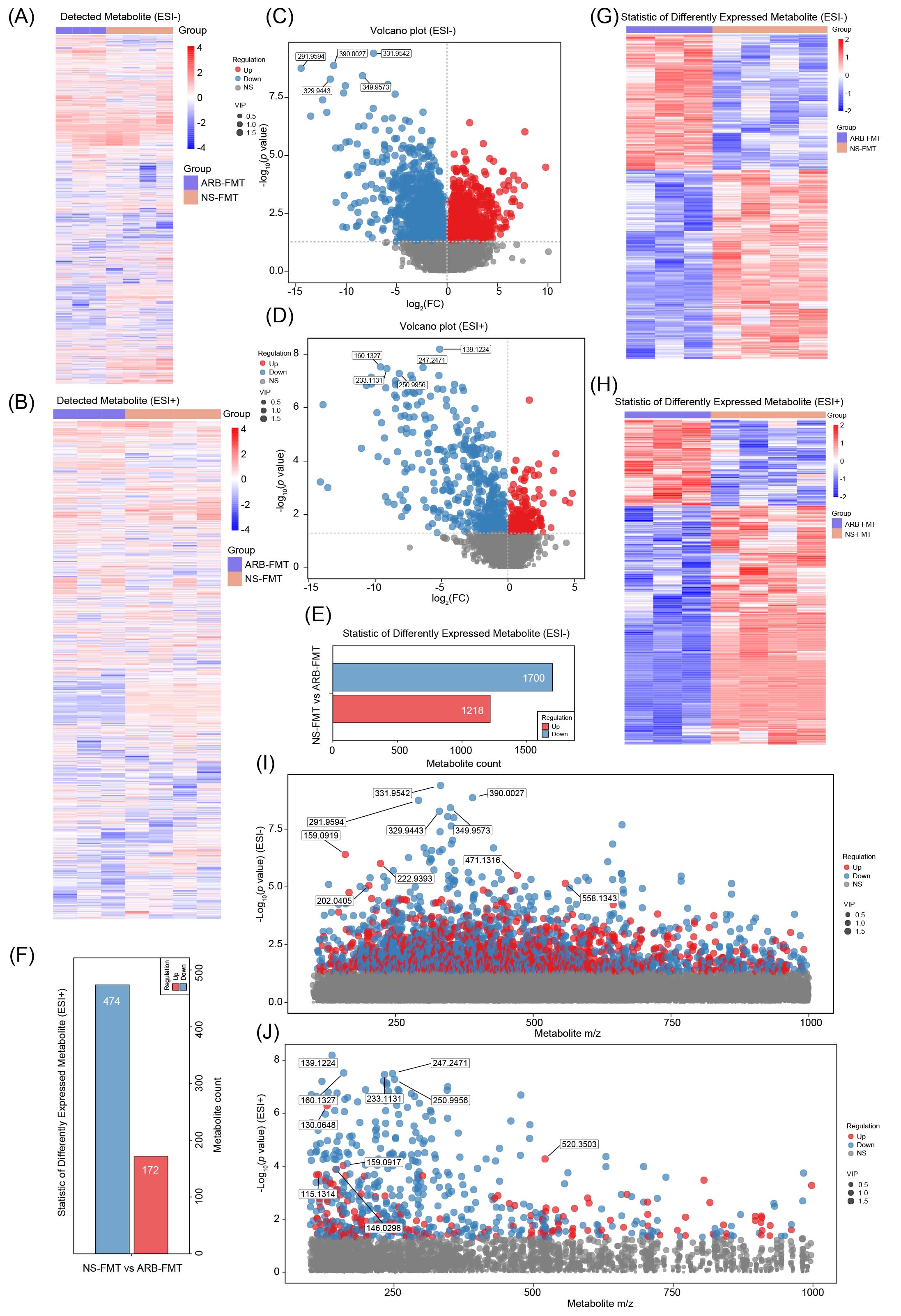
**

Figure S9. Enrichment analysis of serum metabolites detected in NS and valsartan FMT rats. (A-B) Heatmap describing a relative abundance of the serum metabolites in each sample as detected under ESI- and ESI+ modes. The level of metabolites is transformed into *Z* scores by subtracting the average and dividing the standard deviation in all samples. *n* = 4 for NS-FMT, *n* = 3 for ARB-FMT. (C-D) Volcano plots showing the distribution of differential metabolites between ESI- and ESI+ groups. Metabolites with *p* < 0.05, |log_2_FC| > 0, and VIP > 1 are considered to be significantly discriminative. Up in red, compounds markedly abundant in the ARB-FMT group; Down in blue, prominently decreased compounds; NS, compounds not significantly discrepant; FC, Fold Change of ARB-FMT vs. NS-FMT; VIP, variable influence on projection produced by OPLS-DA. Mass-to-charge ratios (m/z) of the top 5 (according to *p*) altered compounds between groups are labeled. (E-F) Bar plots showing the number of significantly disparate compounds when comparing ARB-FMT and NS-FMT samples in ESI- and ESI+ mode. (G-H) Relative abundance of the statistically different abundant metabolites obtained in ESI- and ESI+. (I-J) Scatter plots describing the distribution of *p* value and m/z of detected metabolites in both modes. m/z of the top 5 (according to *p*) enhanced or suppressed compounds in ARB-FMT are labeled respectively.


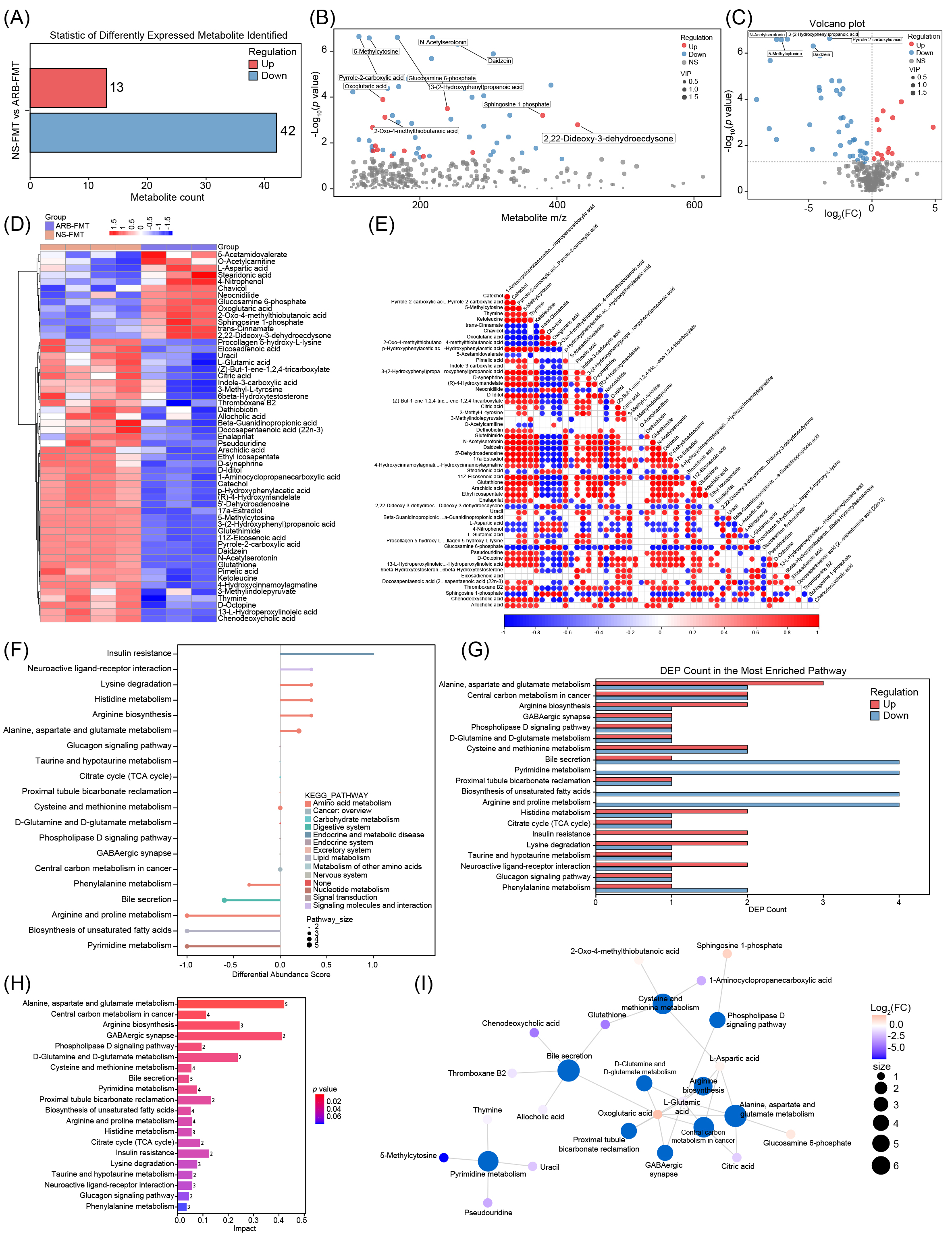


Figure S10. Identification and functional annotation of serum metabolites significantly varied between NS-FMT and ARB-FMT. (A) The number of differently enriched metabolites between groups identified using the Human Metabolome Database, massbank, LipidMaps, and mzcloud. Up/Down, enhanced/decreased in ARB-FMT as compared with NS-FMT. (B) The distribution for *p* value and m/z of metabolites successfully identified is shown in scatter plots. The top 5 metabolites with the lowest *p* value enhanced or suppressed in ARB-FMT are labeled. NS, metabolites not significantly altered; VIP, variable influence on projection produced by OPLS-DA. (C) Volcano plot depicting the distribution of significantly discriminative metabolites between groups. The top 5 most dramatically altered are labeled. FC, Fold Change of ARB-FMT/NS-FMT. (D-E) Relative abundance and correlation pattern of the 55 identified serum metabolites markedly different between NS-FMT and ARB-FMT are visualized under color gradients in heat maps. **F,** The DE serum metabolites identified exert attribution to various KEGG pathways. The differential abundance score shown in the horizontal lollipop chart indicates the number of Up-Down DE metabolites/total compounds within the pathway. Pathway size represents the number of DE metabolites in the pathway. (G-H) Bar plots of DE metabolites count (Up/Down) in the most enriched KEGG pathway and impact index (contribution) of metabolites to the corresponding pathway. *p* values denoted in colors indicate the significance of the influence of metabolites on each pathway. The number of DE metabolites in metabolic pathways is labeled. (I) Correlation network across KEGG pathways and DE metabolites. KEGG pathways are shown in blue, and metabolites are represented in graduated color according to Log_2_(FC); FC, Fold Change. The size of pathway nodes denotes the number of metabolites connected.

**
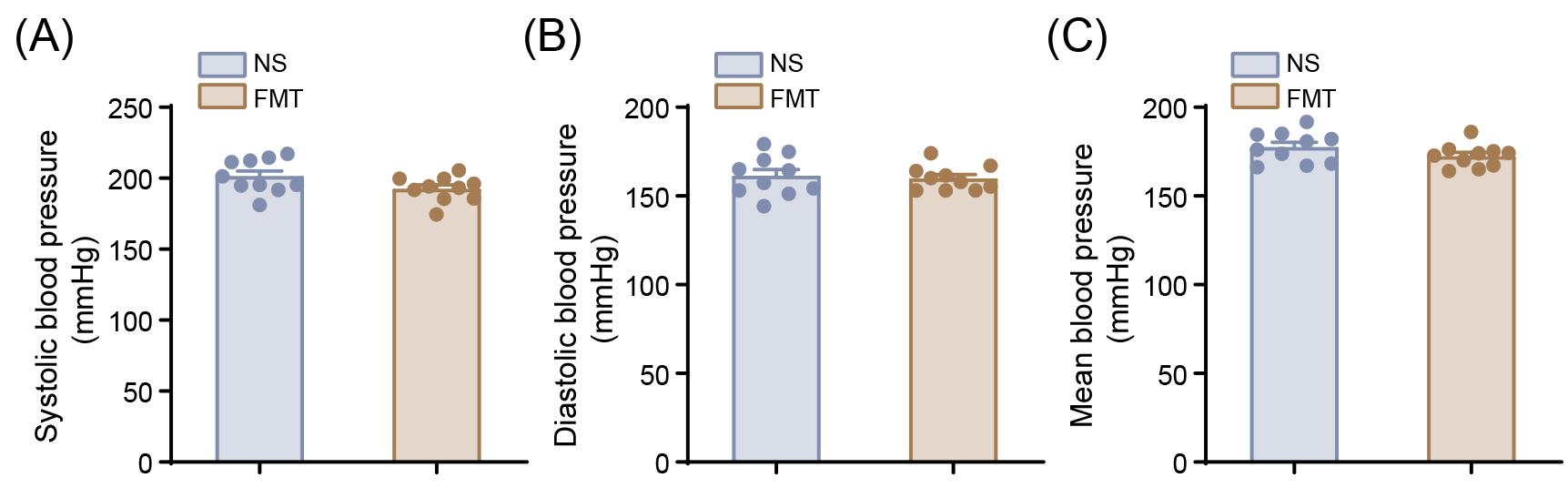
**

Figure S11. Fecal microbiota from hypertensive patients benefiting from ARB treatment exerts inapparent improvement of BP in SHRs. (A-C) Systolic, diastolic, and mean BP in SHRs following NS or FMT administration. *n* = 10/group. Data are presented as mean ± SEM. FMT, intestinal flora transplantation from hypertensive patients with WC hypertension under ARB therapy.

**
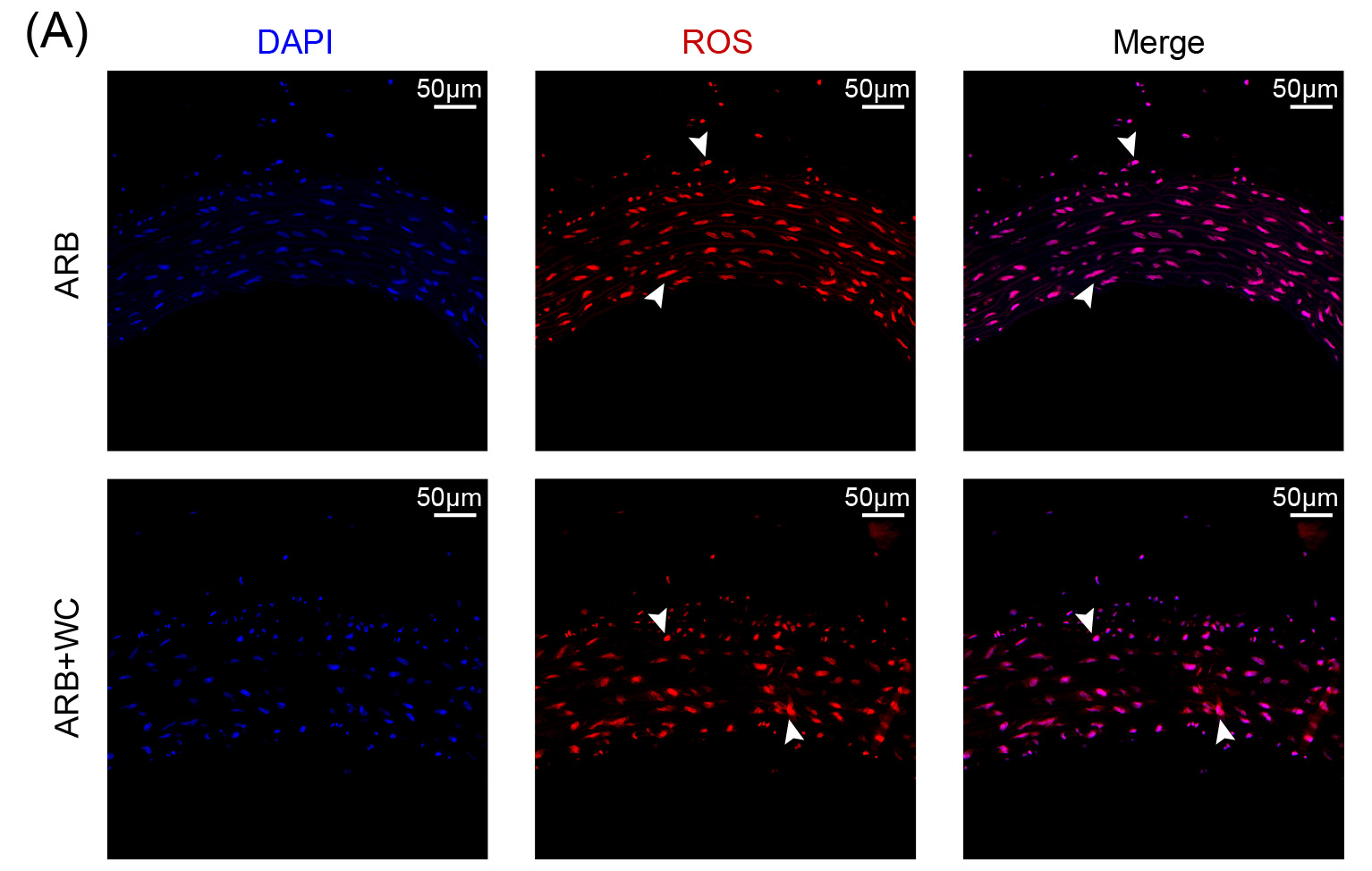
**

Figure S12. Oxidative stress in the vasculature of ARB-treated SHRs is unaffected by gut microbiota from WC hypertensive patients. (A) DHE staining of aortic tissues from valsartan-treated SHRs with or without WC hypertension FMT. Positive red staining was detected, which represents ROS. Blue, nucleus stained with DAPI. The white arrows indicate the ROS positive staining area. DAPI, 4',6-diamidino-2-phenylindole.

**
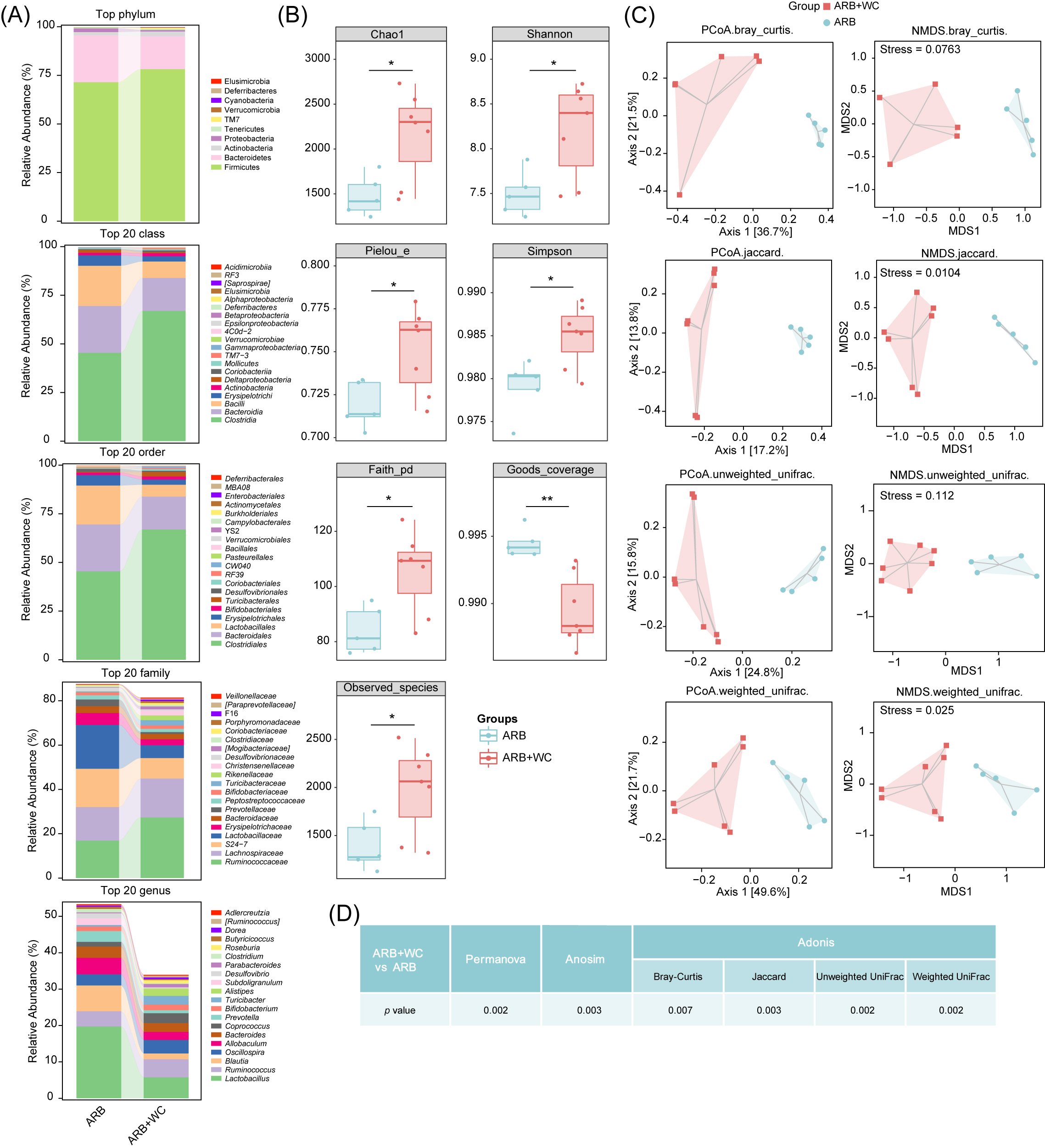
**

Figure S13. Gut flora derived from WC hypertensive patients influence the fecal microbiome of valsartan-treated SHRs. (A) Intestinal microbial profiles in ARB and ARB+WC groups are described with a relative abundance of the most dominant taxonomy at phylum, class, order, family, and genus level, respectively. (B) Alpha diversity parameters of Chao1 richness, Shannon diversity, Pielou evenness, Simpson’s index, Faith’s pd, Goods coverage, and Observed species. *n* = 5 for ARB, *n* = 7 for ARB+WC. **p* < 0.05, ***p* < 0.01; Kruskal-Wallis test. Boxes denote the interquartile ranges, and lines inside represent medians. (C) PCoA and NMDS between groups are conducted based on the Bray Curtis distance, Jaccard distance, unweighted Unifrac distance, and weighted Unifrac distance, respectively. (D) Permanova analysis, anosim analysis, and adonis analysis with Bray Curtis, Jaccard, unweighted Unifrac, and weighted Unifrac distance are performed, and *p* to assess the significance of differences between groups are obtained.

**
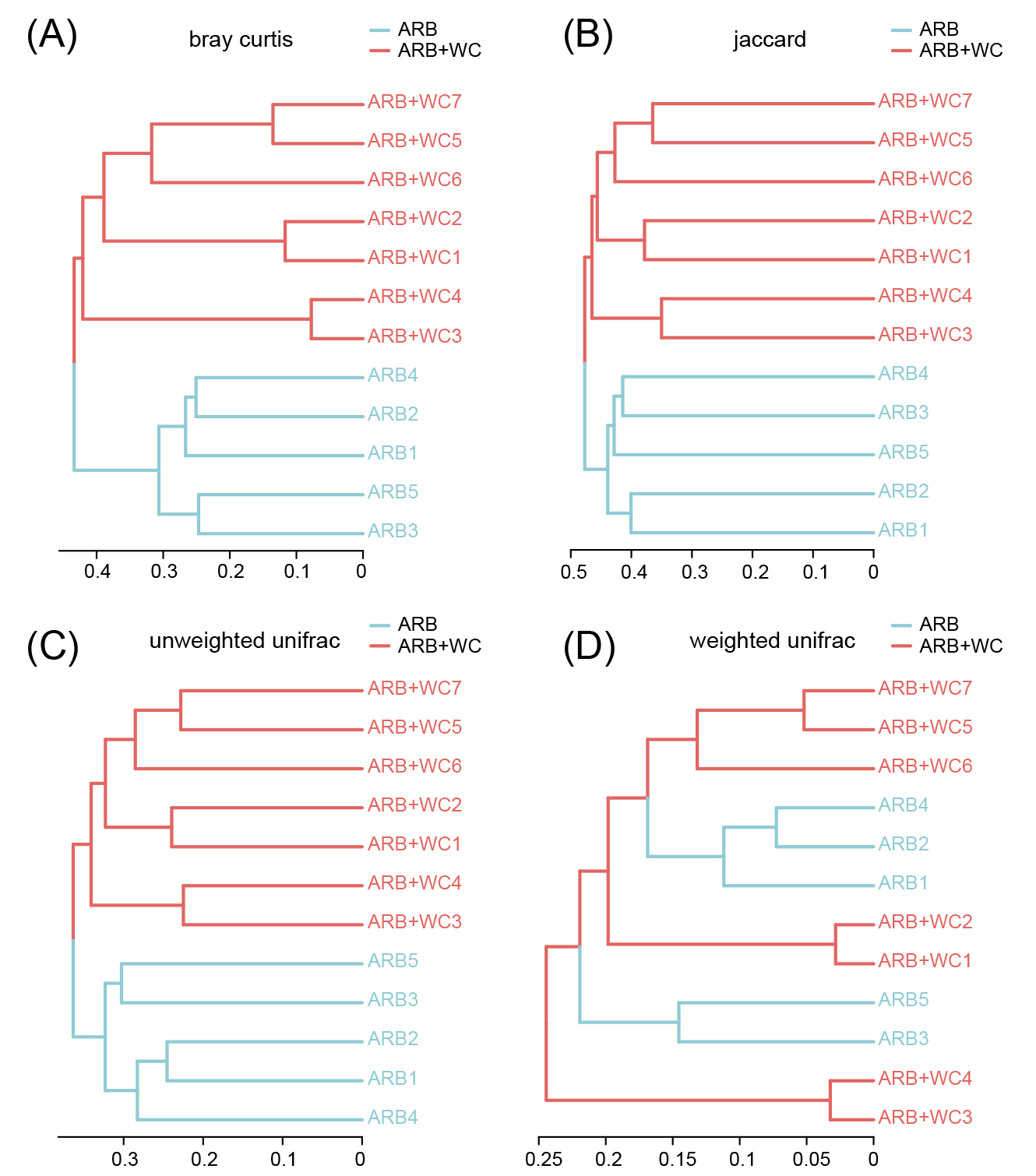
**

Figure S14. Hierarchical cluster of SHRs treated with ARB or ARB+WC according to gut microbial profiles. (A-D) Cluster trees across samples are obtained based on Bray Curtis (A), Jaccard (B), unweighted Unifrac (C), and weighted Unifrac (D) distance using the UPGMA method. The branch length of the clustering tree indicates the effect of clustering.

**
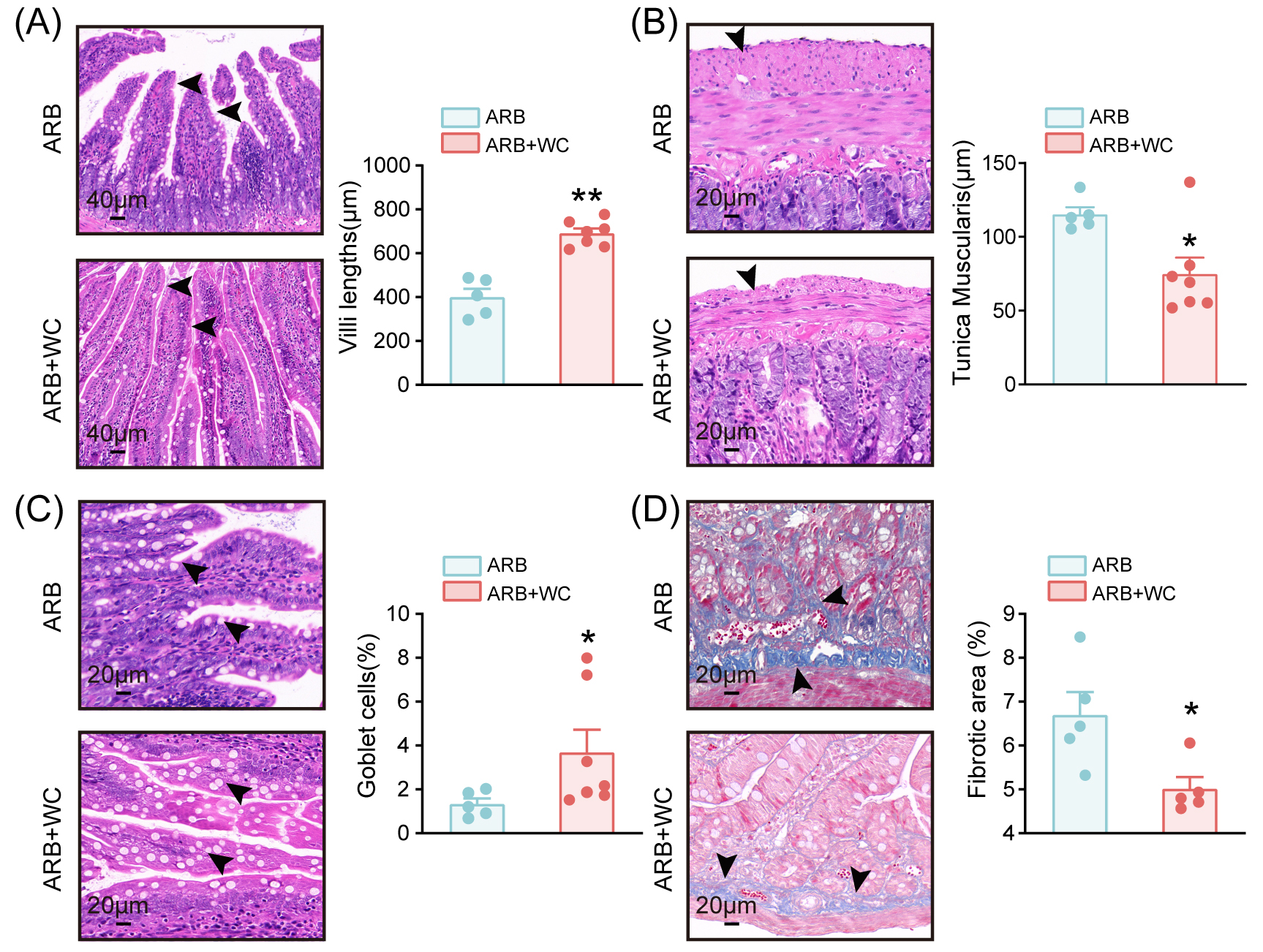
**

Figure S15. Intestinal pathological improvement in ARB-SHRs administrated with WC donors FMT. (A) Cross-sections of the small intestine from valsartan-treated SHRs receiving FMT or not are stained with hematoxylin and eosin. Representative images and quantification of villi lengths in each group of SHRs are shown. The arrows indicate villi. (B) The thickness of the tunica muscularis layer. The arrows denote tunica muscularis. (C) The number of goblet cells per 100 epithelial cells. The arrows indicate goblet cells. *n* = 5 for ARB, *n* = 7 for ARB+WC. (D) The small intestine stained with Masson's trichrome and collagen-positive area is quantified. The arrows point to fibrotic area. *n* = 5 for ARB, *n* = 5 for ARB+WC. Data are presented as mean ± SEM. **p* < 0.05, ***p* < 0.01. Scale bar: 40 μm, and 20 μm.

**
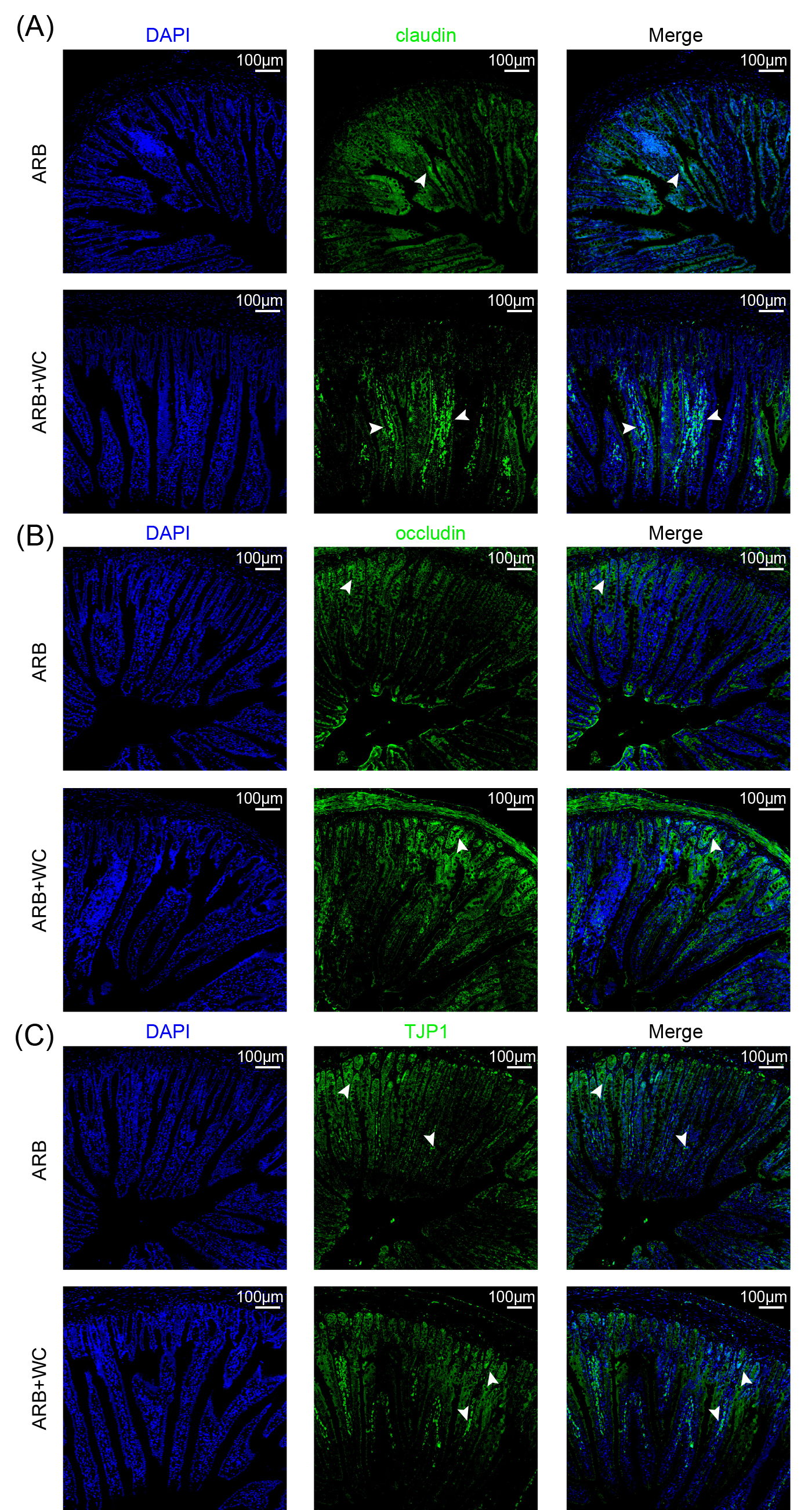
**

Figure S16. Impacts on tight junction proteins in the intestinal tissue by WC fecal microbiota. (A-C) Intestinal tissues of SHRs are stained with antibodies against claudin, occludin, and TJP-1, respectively, and images captured with a fluorescence microscope are displayed. Positive stainings of claudin, occludin, and TJP-1 are dyed in green; nuclei are stained with DAPI in blue. The white arrows point to the immunofluorescence positive staining area. Scale bars are 100 μm.

**
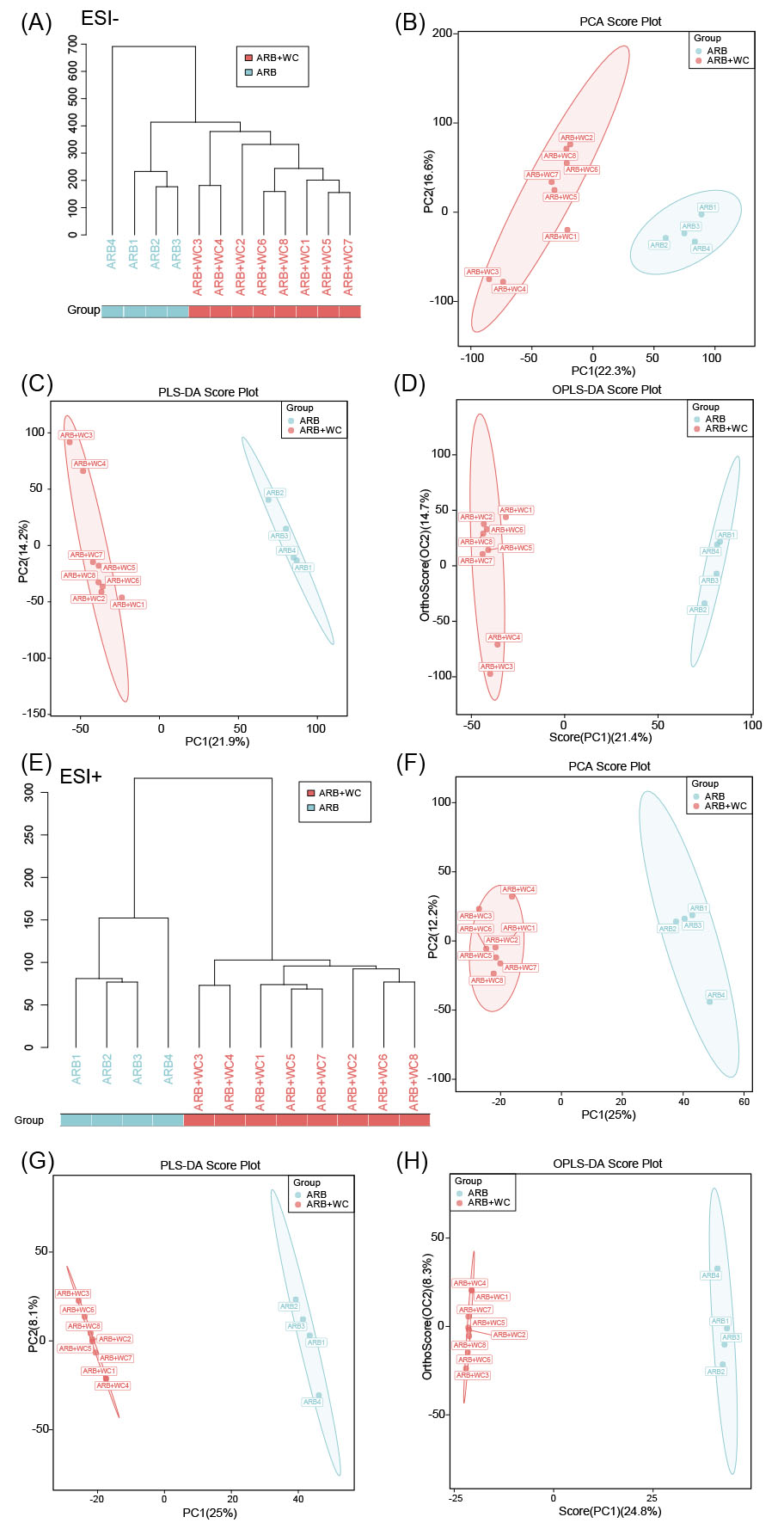
**

Figure S17. Global characteristics of serum metabolome patterns in ARB treated SHRs with and without FMT. (A), (E), Clustering analysis of serum samples according to ESI- and ESI-mode metabolic profiles. Y axis denotes the Euclidean clustering distance. (B-D)**,** (F-H)**,** PCA, PLS-DA, and OPLS-DA in both modes show between-group discrimination. Each component indicates the corresponding explanation degree.

**
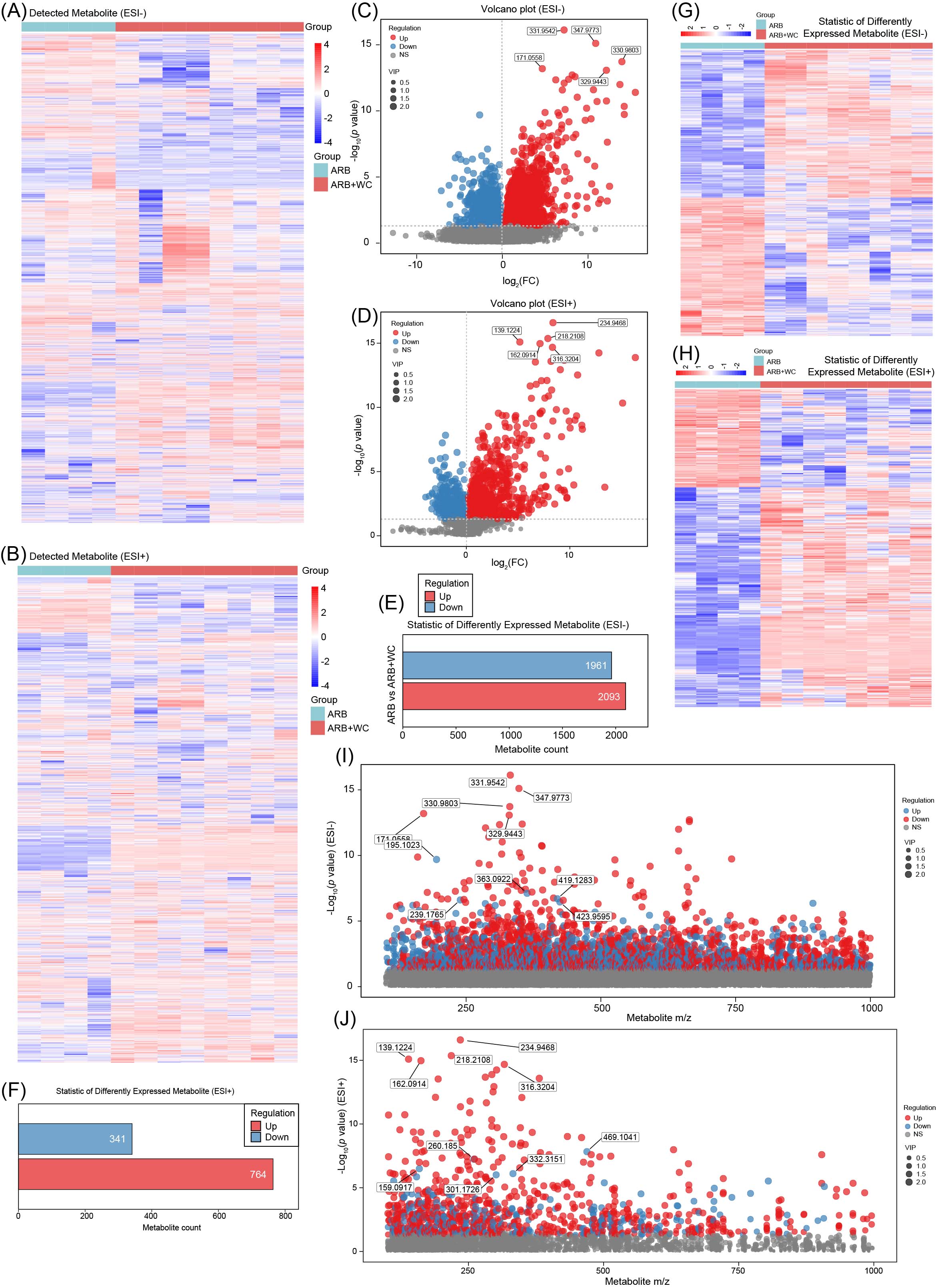
**

Figure S18. Enrichment of serum metabolites detected in ARB and ARB+WC groups. (A-B) Relative abundance of all the detected serum metabolites under ESI- and ESI+ modes in each sample. *n* = 4 for ARB, *n* = 8 for ARB+WC. (C-D) Volcano plots elucidating the distinct metabolites between groups in ESI- and ESI+, respectively. *p* < 0.05, |log_2_FC| > 0 and VIP > 1 are regarded as significantly discrepant. Up/Down, enhanced/reduced in ARB+WC; NS, not significantly different; FC, Fold Change of ARB+WC/ARB. m/z of the top 5 most dramatically distinct compounds is labeled. (E-F) The number of prominently varied compounds in ARB+WC under ESI- and ESI+ mode. (G-H) Relative abundance of the statistically different metabolites between groups. (I-J) The m/z distribution of all detected metabolites in both modes. m/z of the top 5 most dramatically enhanced or reduced compounds in ARB+WC are labeled.


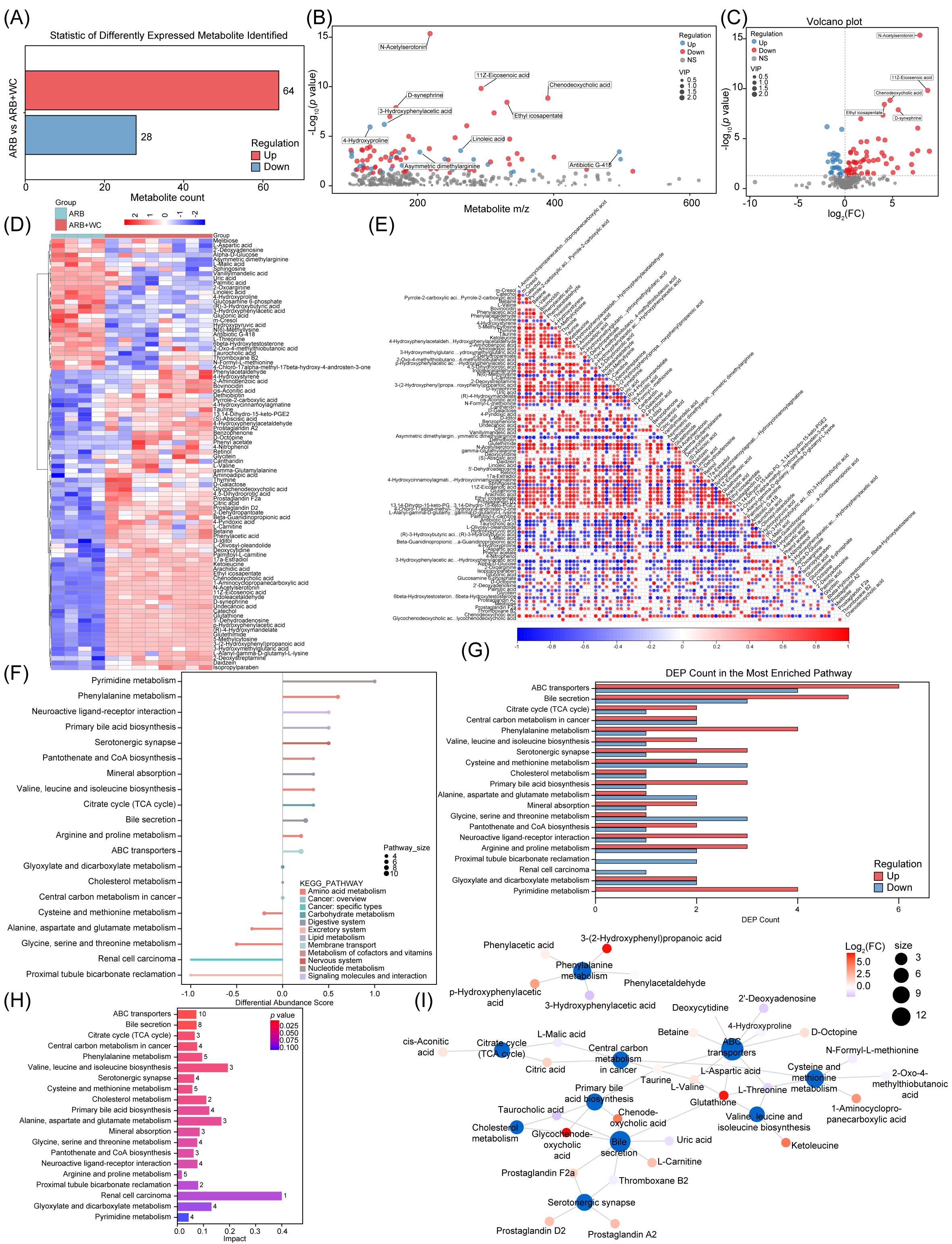


Figure S19. Identification and functional capacity of serum metabolites significantly affected by fecal microbiota from WC donors. (A) The number of significantly enriched (Up) and decreased (Down) metabolites in the ARB+WC group that were successfully identified. (B) The m/z of metabolites identified. The top 5 most obviously enhanced or suppressed metabolites in ARB+WC are labeled. NS, not significantly altered. (C) Volcano plot showing the significantly discriminative metabolites between groups. The top 5 most prominent DE serum metabolites are labeled. FC, Fold Change of ARB+WC/ARB. (D-E) Relative abundance and correlation relationship of the 92 identified serum metabolites dramatically distinct between groups. (F) The DE serum metabolites participate in multiple KEGG pathways. Differential abundance score denotes the number of (Up-Down DE metabolites)/(total compounds within the pathway). Pathway size indicates the number of DE metabolites. (G-H) Count and impact index of DE metabolites in the most enriched KEGG pathways. *p* values denote the significance of the influence of metabolites on pathways. The number represents DE metabolites in each pathway. (I) Network among KEGG pathways and DE metabolites. Pathways are in blue, and metabolites are in colors based on Log_2_(FC); FC, fold change. Node size denotes the number of connecting metabolites.

**
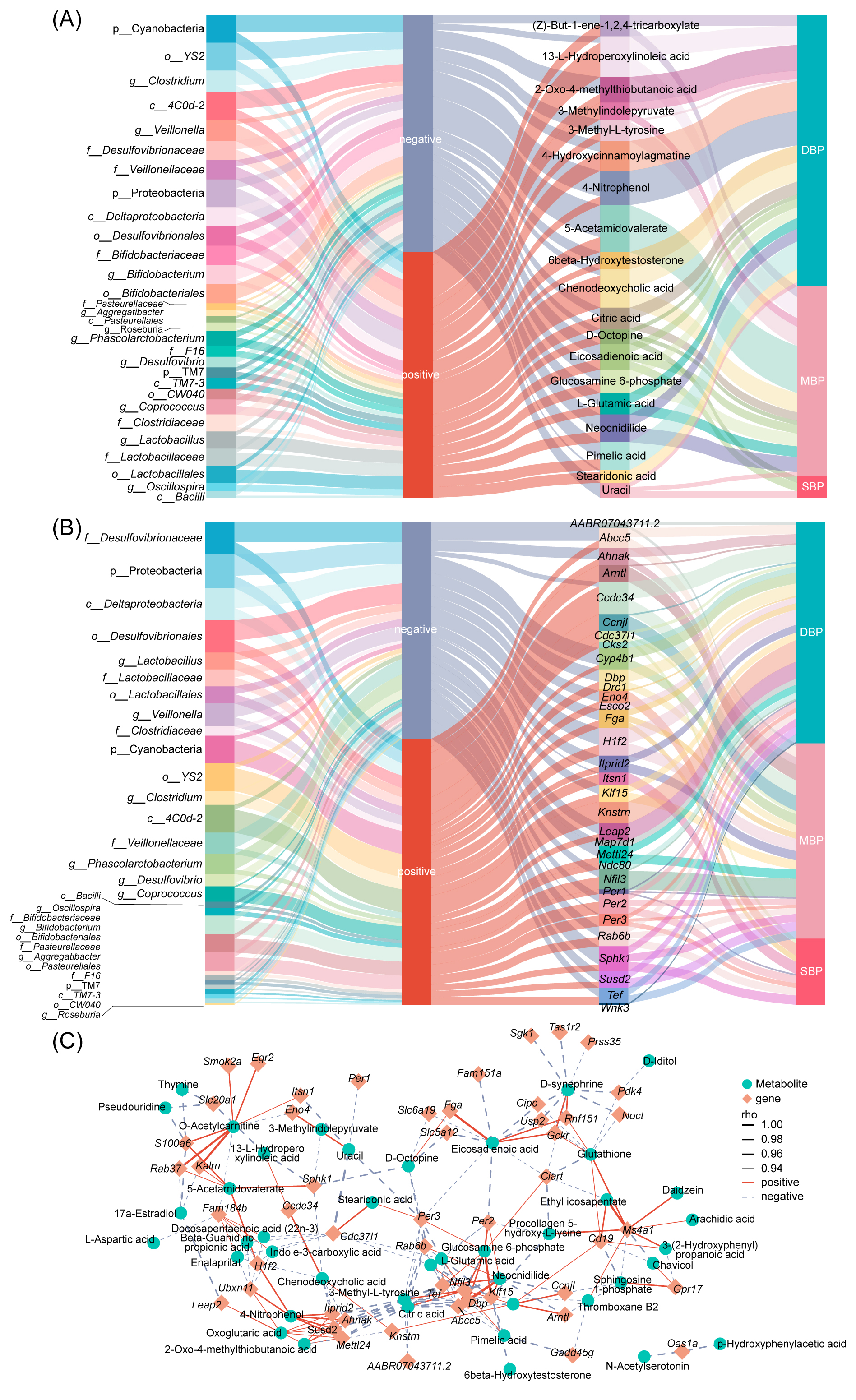
**

Figure S20. Correlation of the microorganisms, serum metabolites, and intestinal gene with hypertension phenotype in SHRs receiving valsartan-microbiota transplantation. (A-B) Interrelationship across the discrepant taxonomy constitution and serum metabolic profiles (A)/transcriptome profiles (B) between SHRs receiving saline or valsartan-treated microbiota and hypertension phenotype was visualized with Sankey diagram based on Spearman correlation analysis. The gut microbes were related to metabolites/genes and further linked to BP. The relation between bacteria and metabolites/genes was depicted with negative (blue) and positive (red), respectively. Each connection indicates a significant correlation with the Spearman correlation test, |*r*| > 0.5, and *p* < 0.05. (C) Co-occurrence network reflecting the interaction between valsartan-related metabolites (green circle) and genes (orange square). Spearmen correlation analysis, |*r*| > 0.9, *p* < 0.01. Lines connecting nodes indicate positive (solid lines in red) or negative (dashed lines in grey) correlations, and the thickness of lines is proportional to the correlation coefficients.

**
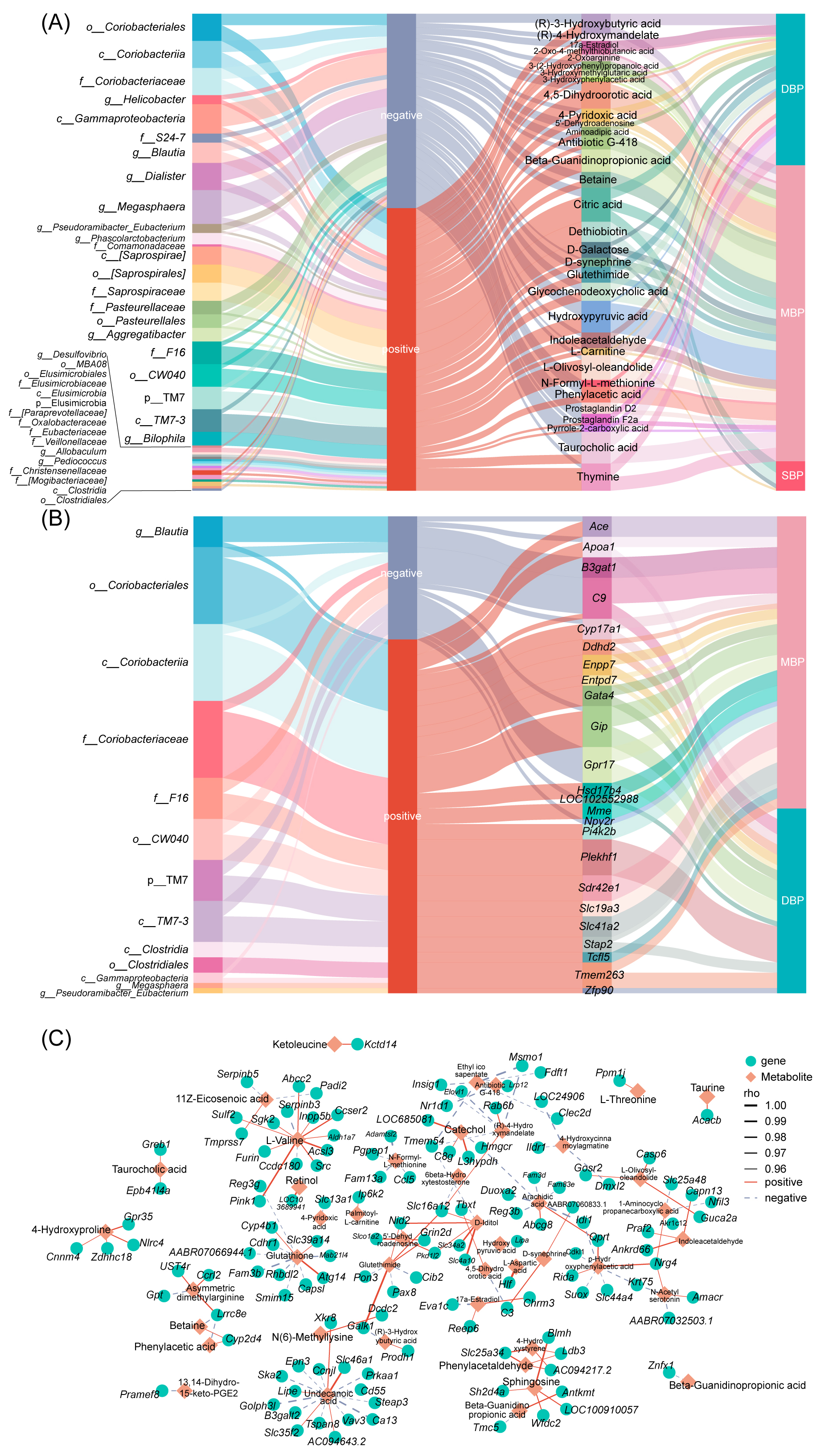
**

Figure S21. Association across the gut microbes, circulating metabolites and intestinal transcriptome, with hypertension phenotype following WC-FMT. (A-B) The relationship between the altered microbial taxon, endogenous metabolites (A) or transcriptional genes (B) in ARB+WC, and hypertension phenotype was estimated by Spearman’s correlation analysis. Sankey diagram illustrated a connection between intestinal flora and serum metabolites/genes associated with BP. Those with low correlation (|*r*| < 0.5, *p* > 0.05) are not shown. Positive and negative correlations are distinguished by color (positive in red and negative in blue). (C) Co-abundance network of the significantly different metabolites (orange square) and genes (green circle) between ARB and ARB+WC. The thresholds derived from Spearman’s correlation analyses are |*r*| > 0.95 and *p* < 0.001. The red solid lines connecting two nodes indicate a positive correlation, and the grey dashed lines represent a negative correlation. The thickness of the line is proportional to the |*r*| value of correlation.
